# Self-consistent theory of transcriptional control in complex regulatory architectures

**DOI:** 10.1371/journal.pone.0179235

**Published:** 2017-07-07

**Authors:** Jasper Landman, Robert C. Brewster, Franz M. Weinert, Rob Phillips, Willem K. Kegel

**Affiliations:** 1 Van ’t Hoff Laboratory for Physical & Colloid Chemistry, Utrecht University, Utrecht, the Netherlands; 2 European Synchrotron Radiation Facility, Grenoble, France; 3 Program in Systems Biology, University of Massachusetts Medical School, Worcester, MA 01605, United States of America; 4 Department of Applied Physics, California Institute of Technology, Pasadena, California, United States of America; 5 Division of Biology and Biological Engineering, California Institute of Technology, Pasadena, California, United States of America; University of California, Davis, UNITED STATES

## Abstract

Individual regulatory proteins are typically charged with the simultaneous regulation of a battery of different genes. As a result, when one of these proteins is limiting, competitive effects have a significant impact on the transcriptional response of the regulated genes. Here we present a general framework for the analysis of any generic regulatory architecture that accounts for the competitive effects of the regulatory environment by isolating these effects into an effective concentration parameter. These predictions are formulated using the grand-canonical ensemble of statistical mechanics and the fold-change in gene expression is predicted as a function of the number of transcription factors, the strength of interactions between the transcription factors and their DNA binding sites, and the effective concentration of the transcription factor. The effective concentration is set by the transcription factor interactions with competing binding sites within the cell and is determined self-consistently. Using this approach, we analyze regulatory architectures in the grand-canonical ensemble ranging from simple repression and simple activation to scenarios that include repression mediated by DNA looping of distal regulatory sites. It is demonstrated that all the canonical expressions previously derived in the case of an isolated, non-competing gene, can be generalised by a simple substitution to their grand canonical counterpart, which allows for simple intuitive incorporation of the influence of multiple competing transcription factor binding sites. As an example of the strength of this approach, we build on these results to present an analytical description of transcriptional regulation of the *lac* operon.

## Introduction

Transcriptional regulation is essential for shaping cellular response and dynamics. At the heart of these responses is the specific arrangement of regulatory features around the promoter that governs how a gene will respond to the available regulatory molecules [1]. A primary goal in the field of systems biology is to elucidate the rules governing how regulation is encoded in the DNA enabling a bottom-up approach to designing regulatory architectures and understanding cellular physiology. A necessary step towards this goal is the development of detailed, predictive theory that takes as input the regulatory architecture (how the regulatory features are arranged on the DNA) and the nature of the regulatory environment and yields a prediction for the level of transcriptional output.

Statistical mechanical models have been used to quantitatively describe transcriptional regulation for a variety of regulatory motifs [2–17]. In those models, the activity of a gene is assumed to be proportional to the probability of an RNA-polymerase (RNAP) being bound to the promoter sequence. This is a precondition for the subsequent initiation of the transcription process, which ultimately leads to the production of proteins [18–21]. However, the equilibrium assumptions needed to treat transcription regulation in this quasi-static limit are subtle. There exists a corresponding class of models that are based on kinetics and therefore do not require as many assumptions, at the cost of increasing the number of parameters that are required [22–28]. In both classes of models, transcription factors can bind to specific binding sites on the DNA and regulate transcription, often by interacting with the RNAP and altering its probability to bind to the promoter. The magnitude of transcriptional regulation is typically quantified as the fold-change in gene expression (fold-change), defined as the level of gene expression in the presence of those transcription factors divided by the level of gene expression in the absence of the transcription factors.

While statistical mechanical models of gene expression have thus far proven to be very successful, they have traditionally been derived in the “non-interacting” limit, i.e. the gene of interest is treated as being isolated and the relevant molecules only interact with the gene itself and a competing “non-specific reservoir” accounting for the generic interaction between the molecules and the rest of the genome [8, 10, 29, 30]. However, in most cases transcription factors act on multiple different genes and as a consequence, the number of available transcription factors can be substantially reduced due to binding at those genes (see e.g. [31] Fig 3b). In addition, multiple copies of the same gene may exist within one cell, for example in the form of duplicate chromosomes, plasmids or viral DNA. Several theoretical efforts have explored the consequences of the titration effect considered here [32–36]. The impact of these competitive interactions can be accounted for in the canonical ensemble using combinatorics to keep track of the possible arrangements of transcription factors to an arbitrary arrangement of binding sites, however the resulting predictions do not lend themselves to simple intuitive interpretation [17, 34, 37]. We have recently shown that a formalism based on the grand-canonical ensemble provides a clear and straightforward interpretation of the impact of transcription factor sharing for one particular regulatory architecture [38]. Our model leads to a simple analytical expression for the fold-change that is in excellent agreement with the available experimental data.

In this work we go well beyond these earlier efforts to show that the grand-canonical approach can be generalized to include more complex regulatory architectures, opening the door to considering regulation in the setting of real cellular processes. Specifically, we demonstrate how to derive expressions for the fold-change for regulatory architectures that have not previously been described using this formalism, including how to characterize such architectures in the case of multiple gene copies and competing reservoirs for transcription factors. Interestingly, all grand-canonical expressions that are derived in this work differ from their corresponding canonical expressions merely by a simple substitution.

In the remainder of this article, we derive expressions for the fold-change for regulatory architectures that include repression, activation and repression by DNA looping, and show how to combine different regulatory elements into more complex architectures. We will provide, as a case study, a worked example for the fold change in expression from the *lac* operon, a regulatory architecture that includes repression, both through proximal binding and by DNA looping interactions, and activation. Table 1 shows an overview of the notation used in this work.

**Table 1:** Summary of notation used in this work.

Symbol	Explanation
*λ*_*m*_	Fugacity of transcription factor *m*
*ϵ*_*m*_	Adsorption energy of transcription factor *m* to its specific site
*x*_*m*_	exp(−*βϵ*_*m*_)
*θ*_*m*_	Average occupation of transcription factor *m* on its specific site
$\epsilon_{m}^{n}$	Adsorption energy of transcription factor *m* to site *n*
$x_{m}^{n}$	$\exp(-\beta \epsilon_{m}^{n})$
$\theta_{m}^{n}$	Average occupation of transcription factor *m* to site *n*
$F_{\text{L}}^{ab}$	Free energy of forming a loop between sites *a* and b*
$x_{\text{L}}^{ab}$	$\exp(-\beta F_{\text{L}}^{ab})$
$\theta_{m}^{ab}$	Average occupation of transcription factor *m*, adsorbed to sites *a* and *b*, forming a loop
$\Delta F_{m\text{L}}^{ab}$	Change in the looping free energy between sites *a* and *b*, due to the presence of transcription factor *m*
$x_{m\text{L}}^{ab}$	$\exp(-\beta \Delta F_{m\text{L}}^{ab})$
*P*, *R*, *A*	Number of RNAP, repressor or activator molecules in the cell
*N*	Gene copy number
*N*_ns_	Number of non-specific sites on the DNA
Ξ	Grand canonical partition function
*Z*	Relevant part of the canonical partition function

*The superscript has been dropped if the loop between sites *a* and *b* is the only possible loop

## Repression architectures

### Simple repression

Transcription initiation is a complex process involving multiple steps, each with their own rate. In its most simplified form, it can be described in three steps: the binding of RNAP to the promoter to form a closed complex, the (irreversible) isomerisation of the closed complex to an open complex, followed by the escape of the open complex to form an RNAP complex active in transcription [18–21]. When the rearrangement of RNAP and transcription factors is fast compared to the formation of an open complex, we can assume that the rate at which the open complex is formed—the first kinetically significant step in the transcription process—is proportional to the occupation probability of the promoter by RNAP. The applicability of this approximation has to be considered on a case by case basis, as there is evidence for slow transcription factor binding and unbinding kinetics in some organisms and circumstances [39–43].

Statistical mechanics provides the tools to calculate the occupation probability of RNAP and transcription factor binding sites, where the RNAP and transcription factors are shared between many different binding sites. The ensemble of choice for a system where the number of molecules is allowed to fluctuate is the grand canonical ensemble. While strictly most suitable for systems with large numbers of particles, the relative fluctuations decrease quickly as $\sigma /\langle N\rangle =1/\sqrt{N}$, as the number of particles grows [44]. We therefore consider the gene of interest as a grand canonical system that is decoupled from the rest of the genome, which acts as the reservoir. The system is kept in equilibrium with reservoirs for all other types of binding site, characterised by a constant chemical potential of the proteins. Each reservoir of a certain type of binding site is considered an independent grand canonical system in its own right. The chemical potential is then found self-consistently by application of the appropriate boundary condition, namely, the conservation of the number of proteins in the cell. The transcription factors that we consider generally have a very high affinity for DNA, even outside of its specific binding site [1, 45]. Consequently, the fraction of transcription factors that is not adsorbed to any DNA site can usually be neglected, as we have shown in our previous work [38]. We have chosen to measure all binding energies with respect to the binding energy of proteins to the non-specific genomic background. An added complication here is that not every non-specific site has an equal binding energy. In first approximation the occupation of a reservoir with a Gaussian distribution of binding energies is equal to that of a reservoir of identical sites with a binding energy of 〈*ϵ*^ns^〉 − *βσ*^2^/2 with 〈*ϵ*^ns^〉, *σ* the mean and standard deviation, respectively, of the distribution of binding energies to non-specific sites and *β* = (*k*_B_*T*)^−1^ [46]. All energies in this work are given relative to this reference energy.

**Fig 1:**
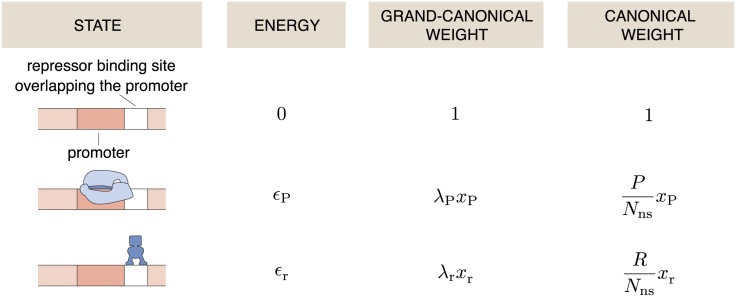
States and their weights in the simple repression architecture. All allowed states of the simple repression architecture are shown with their associated energies and statistical weights. *ϵ*_P_ is the binding energy of the RNAP onto the promoter site, *ϵ*_R_ the binding energy of a repressor molecule onto the operator site. The third column shows the grand canonical weights, where the *λ*_*i*_ is the fugacity of the RNAPs (*i* = P) or repressors (*i* = R), and $x_{i}=e^{-\epsilon_{i}/k_{\text{B}}T}$. The right column lists the weights in the canonical ensemble where *P* is the number of RNA-polymerase molecules, *R* the number of repressors, and *N*_ns_ the number of non-specific binding sites of the genome.

We start with the simplest nontrivial regulatory architecture referred to as ‘simple repression,’ as illustrated in the first column in Fig 1. This architecture consists of a promoter and an operator site for a repressor molecule. An RNAP can bind to the promoter with binding energy *ϵ*_P_, and a repressor can bind to the operator site with energy *ϵ*_R_, while the simultaneous binding of both, RNAP and repressor is prohibited by excluded-volume interactions.

The grand canonical partition function [38] of a single gene with this regulatory architecture unit is given by

$$\begin{array}{c} \Xi =\sum_{p=0}^{1}\sum_{r=0}^{1-p}\lambda_{\text{P}}^{p}\lambda_{\text{R}}^{r}Z\left(p,r\right)=1+\lambda_{\text{P}}x_{\text{P}}+\lambda_{\text{R}}x_{\text{R}}, \end{array}$$ (1)

where the fugacity of a repressor molecule is given by $\lambda_{\text{R}}=e^{\beta \mu_{\text{R}}}$, where *μ*_R_ is the chemical potential of a repressor molecule. Similarly $\lambda_{\text{P}}=e^{\beta \mu_{\text{P}}}$ where *μ*_P_ is the chemical potential of an RNAP molecule. The indices *p* and *r* reflect the number of RNAP and repressor molecules, respectively, that are bound to the gene in a given occupational state with *Z*(*p*, *r*) the relevant part of the canonical partition function. The factor *Z* is given by the product of all the Boltzmann exponents of the individual binding free energies of the DNA-bound transcription factors, and of the interactions that take place between them when they are bound in that arrangement. All other internal degrees of freedom remain constant, and therefore do not contribute to the weight of a configurational state. This modular approach allows the framework to be used in conjunction with automated scripts to calculate the statistical weight of a configurational state. While for simple promoter architectures it is possible to write down the statistical weights for all the individual configurational states, this quickly becomes cumbersome when the complexity of the promoter architecture increases. Similar state-weight scripts have been demonstrated for models based on the canonical ensemble, for example in ref [47].

For the motif of simple repression *Z*(0, 0) = 1, $Z\left(1,0\right)=e^{-\beta \epsilon_{\text{P}}}=x_{\text{P}}$ and $Z\left(0,1\right)=e^{-\beta \epsilon_{\text{R}}}=x_{\text{R}}$. Binding of both RNAP and repressor is prohibited by excluded volume interactions, effectively meaning that the weight of *Z*(1, 1) is zero and that term is excluded in Eq (1). In the case of *N* statistically independent gene copies we have Ξ_s_ = Ξ^*N*^ as in our previous work (Eq (2) in [38]). It can immediately be checked that Ξ is given by adding up the weights in the third column in Fig 1. Similarly, the relevant canonical partition function in [4, 7, 8] is given by adding up the weights in the right-hand column in Fig 1.

The fraction of binding sites occupied by its cognate transcription factor is calculated by [48]

$$\begin{array}{c} \theta_{i}=\frac{1}{N}\frac{\partial \log\Xi_{\text{s}}}{\partial \log\lambda_{i}}, \end{array}$$ (2)

with *λ*_*i*_ the fugacity of the cognate transcription factor *i*. Since all *N* gene copies are independent and identical, the occupational fraction *θ*_*i*_ can (and will in the remainder of this work) also be calculated from the single gene partition function Ξ from the easier, but mathematically equivalent equation

$$\begin{array}{c} \theta_{i}=\frac{\lambda_{i}}{\Xi }\frac{\partial \Xi }{\partial \lambda_{i}}. \end{array}$$ (3)

Fold-change, defined as the gene expression in the presence of transcription factors divided by gene expression in the absence of transcription factors, can be calculated as fraction of promoters occupied by RNAP in the presence of repressors normalized by the fraction of RNAP occupied promoters the absence of repressor. In the presence of transcription factors, this fraction becomes,

$$\begin{array}{c} \theta_{\text{P}}\left(\lambda_{\text{P}},\lambda_{\text{R}}\right)=\frac{\lambda_{\text{P}}}{\Xi }\frac{\partial \Xi }{\partial \lambda_{\text{P}}}=\frac{\lambda_{\text{P}}x_{\text{P}}}{1+\lambda_{\text{P}}x_{\text{P}}+\lambda_{\text{R}}x_{\text{R}}}. \end{array}$$ (4)

In the absence of repressors we have

$$\begin{array}{c} \theta_{\text{P}}\left(\lambda_{\text{P}},0\right)=\frac{\lambda_{\text{P}}x_{\text{P}}}{1+\lambda_{\text{P}}x_{\text{P}}}. \end{array}$$ (5)

In writing down Eqs (4) and (5), we assumed that the fugacities *λ*_P_, *λ*_R_ are independent, that is, the value of *λ*_P_ does not depend on the repressor concentration (or fugacity). As shown in the supporting information (see S1 Text), this is an excellent approximation for all the cases considered here. Fold-change is now given by

$$\begin{array}{c} \text{fold-change}=\frac{\theta_{\text{P}}\left(\lambda_{\text{P}},\lambda_{\text{R}}\right)}{\theta_{\text{P}}\left(\lambda_{\text{P}},0\right)}=\frac{1+\lambda_{\text{P}}x_{\text{P}}}{1+\lambda_{\text{P}}x_{\text{P}}+\lambda_{\text{R}}x_{\text{R}}}. \end{array}$$ (6)

Using the grand canonical ensemble, we have essentially decoupled the individual gene copies from each other and the rest of the genome. With the system in quasi-static equilibrium with non-regulatory and other competing reservoirs, the chemical potential of the RNAP and repressors is equal in all binding reservoirs. Therefore, we can obtain the values of the fugacities *λ*_P_, *λ*_R_ self-consistently by applying the appropriate boundary conditions, that is, conservation of the total number of RNAP and repressors in a cell.

In general, the molecules can bind to their specific binding sites related to *N* ≥ 1 copies of the gene of interest, to the reservoir of *N*_ns_ ≫ 1 non-specific binding sites, or to a set of *i* ≥ 0 additional reservoirs, each with *N*_*i*_ binding sites, which can be binding sites related to competitor genes. Individual molecules can transfer between reservoirs, while the total number of molecules in the cell is conserved. When needed, a reservoir for free transcription factors can be included. However, as mentioned before, the fraction of transcription factors unbound to DNA is generally negligible, hence our choice is to not to include a reservoir for free transcription factors in solution. The fugacities *λ*_P_, *λ*_R_ are set by the constraint that mass is conserved inside the cell, and can be found by setting up a mass balance that contains all relevant reservoirs. For repressors, *λ*_R_ follows from

$$\begin{array}{c} R=N\theta_{\text{R}}+N_{\text{ns}}\theta_{\text{R}}^{\text{ns}}+\sum_{i}N_{i}\theta_{\text{R}}^{i}, \end{array}$$ (7)

with $\theta_{\text{R}},\theta_{\text{R}}^{\text{ns}}$ and $\theta_{\text{R}}^{i}$ being the repressor bound fraction of specific sites, non-specific sites and sites belonging to any additonal reservoir *i*, respectively. If we have a set *j* containing additional reservoirs for RNAP, each with *N*_*j*_ binding sites, the value of *λ*_P_ follows similarly from

$$\begin{array}{c} P=N\theta_{\text{P}}+N_{\text{ns}}\theta_{\text{P}}^{\text{ns}}+\sum_{j}N_{j}\theta_{\text{P}}^{j}, \end{array}$$ (8)

now with $\theta_{\text{P}},\theta_{\text{P}}^{\text{ns}}$ and $\theta_{\text{P}}^{j}$ being the RNAP bound fraction of specific sites, non-specific sites and sites belonging to reservoir *j*, respectively.

In the rather general situation that *λ*_P_*x*_P_ ≪ 1, referred to as the weak promoter limit, we have

$$\begin{array}{c} \text{fold-change}=\frac{1}{1+\lambda_{\text{R}}x_{\text{R}}},\;\left(\lambda_{\text{P}}x_{\text{P}}\ll 1\right) \end{array}$$ (9)

which is exactly the result in [38]. Unless stated otherwise, we will focus in this work on the weak-promoter limit, yet in all the contexts that follow it is straightforward to consider the more general limit where the inequality in parenthesis in Eq (9) does not apply. In the weak promoter limit there is only a single conservation relation to be solved, that is, conservation of repressor, in order to obtain the value of *λ*_R_. In this limit, *θ*_R_ follows from Eq (1) where *λ*_P_*x*_P_ ≪ 1, i.e.

$$\begin{array}{c} \theta_{\text{R}}\left(\lambda_{\text{R}}\right)=\frac{\lambda_{\text{R}}x_{\text{R}}}{1+\lambda_{\text{P}}x_{\text{P}}+\lambda_{\text{R}}x_{\text{R}}}≃\frac{\lambda_{\text{R}}x_{\text{R}}}{1+\lambda_{\text{R}}x_{\text{R}}}.\;\left(\lambda_{\text{P}}x_{\text{P}}\ll 1\right) \end{array}$$ (10)

Interestingly, solving for an isolated promoter in the canonical ensemble using the states and weights in the right hand column in Fig 1, results in [7, 8, 37, 45]

$$\begin{array}{c} \text{fold-change}=\frac{1}{1+\left(\frac{R}{N_{\text{ns}}}\right)x_{\text{R}}}.\;\left(\text{canonical}\right) \end{array}$$ (11)

The similarity between Eqs (9) and (11) implies that in order to obtain an expression for the fold-change that is valid for any number of gene copies, additional binding sites, etc, we may simply take the canonical, single-gene result and replace *R*/*N*_ns_ by *λ*_R_. This proves to be the case for any regulatory architecture, as we show in the supporting information (see S5 Text).

In the limit that 1 ≪ *R* ≪ *N*_ns_, the canonical and grand-canonical expressions become equivalent. To see that, consider the average number of repressors bound to non-specific sites, which is given by

$$\begin{array}{c} \left\langle R_{\text{ns}}\right\rangle =N_{\text{ns}}\theta_{\text{R}}^{\text{ns}}=N_{\text{ns}}\frac{\lambda_{\text{R}}x_{\text{R}}^{\text{ns}}}{1+\lambda_{\text{R}}x_{\text{R}}^{\text{ns}}}\approx N_{\text{ns}}\lambda_{\text{R}}. \end{array}$$ (12)

Since we have set the reference point of energy to the binding energy of repressors to non-specific sites as discussed above, $x_{\text{R}}^{\text{ns}}=e^{0}=1$, and we took *λ*_R_ ≪ 1 which is valid as long as *R* ≪ *N*_ns_ [38]. Thus, we have *λ*_R_ = 〈*R*_ns_〉/*N*_ns_, which, for a single gene copy per cell, asymptotically approaches *R*/*N*_ns_ at large *R*. While not exact for small *R*, *λ*_R_ ≈ *R*/*N*_ns_ is a good approximation in most physiological situations (again with a single gene per cell) where cells typically contain multiple repressor copies.

In the remainder of the paper we show that more complicated regulatory architectures that have been analyzed in the canonical ensemble [7, 8] can easily be translated into the grand-canonical formalism making it possible to calculate fold-change for the cases of multiple gene copies or competing binding sites.

### Repression with looping

Though it is one of the most common architectures, the simple repression regulatory motif described above is only one of many common regulatory motifs [49, 50]. In the following section we consider the impact of transcription factors with two DNA binding domains that are capable of binding two operator sites simultaneously. These auxiliary operator sites can enhance the efficacy of the transcription factor by increasing the probability of occupancy of the main operator site, where it is able to regulate transcription, by allowing for loops in the DNA between the operator sites. Thus we must take into account both the energetic benefit to the system from binding an extra operator weighed against the free energy penalty associated with the reduced configurational freedom of the DNA [51].

**Fig 2:**
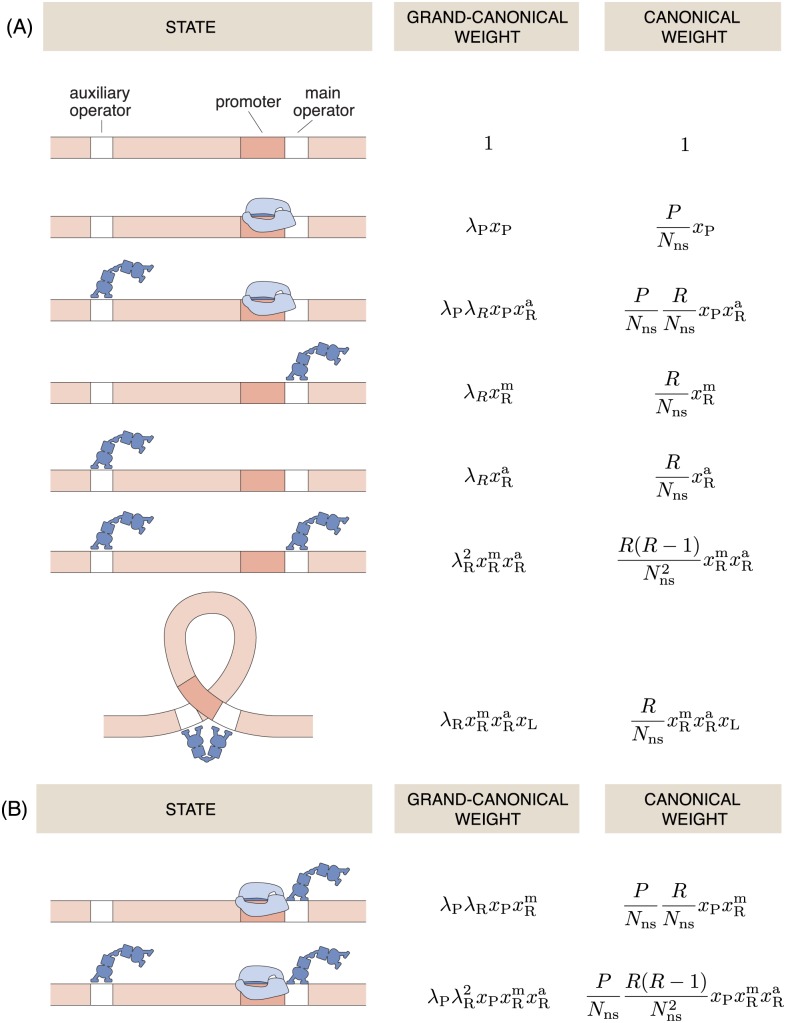
Grand canonical states and weights in the looping architecture. (**A**) Looping architecture where a repressor bound to the main operator and RNAP binding are mutually exclusive. (**B**) Additional states and weights for the exclusive looping scenario. In this scenario, repression is only effective in the looped state.

Consider *N* copies of a gene that contains a main and an auxiliary operator site, denoted by m and a, respectively, and a promoter site P for RNAP. The architecture as well as a table of states and weights is shown in Fig 2A. The grand partition function of a single copy of this regulatory unit reads

$$\begin{array}{ccc} \Xi & = & \sum_{p=0}^{1}\sum_{r=0}^{2}\lambda_{\text{P}}^{p}\lambda_{\text{R}}^{r}Z\left(p,r\right)\\ & = & 1+\lambda_{\text{P}}x_{\text{P}}+\lambda_{\text{R}}\left(x_{\text{R}}^{\text{a}}+x_{\text{R}}^{\text{m}}+x_{\text{R}}^{\text{a}}x_{\text{R}}^{\text{m}}x_{\text{L}}\right)+\lambda_{\text{P}}\lambda_{\text{R}}x_{\text{R}}^{\text{a}}x_{\text{P}}+\lambda_{\text{R}}^{2}x_{\text{R}}^{\text{a}}x_{\text{R}}^{\text{m}}, \end{array}$$ (13)

where the fugacities have been defined below Eq (1). *Z*(*p*, *r*) is the relevant measure of the canonical partition function when *p* RNAP molecules and *r* repressors are adsorbed onto the promoter region. Just as in the case of simple repression above, configurations that include a repressor bound to the main site and an RNAP bound to the promoter simultaneously are given zero weight. Further we define $x_{\text{P}}=e^{-\beta \epsilon_{\text{P}}}$, $x_{\text{R}}^{\text{a}}=e^{-\beta \epsilon_{\text{R}}^{\text{a}}}$, $x_{\text{R}}^{\text{m}}=e^{-\beta \epsilon_{\text{R}}^{\text{m}}}$ with *ϵ*_P_, $\epsilon_{\text{R}}^{\text{a}}$, and $\epsilon_{\text{R}}^{\text{m}}$ the binding energy of RNAP to a promoter site, and the repressor to an auxiliary site and to a main site, respectively. In addition, we define $x_{\text{L}}=e^{-\beta F_{\text{L}}}$ where *F*_L_ is the free energy cost associated with forming a loop. In writing down the right side of Eq (13) we used for *Z*(*p*, *r*):

$$\begin{array}{c} Z\left(0,0\right)=1,\;Z\left(1,0\right)=x_{\text{P}},\;Z\left(0,1\right)=x_{\text{R}}^{\text{a}}+x_{\text{R}}^{\text{m}}+x_{\text{R}}^{\text{a}}x_{\text{R}}^{\text{m}}x_{\text{L}},\\ Z\left(1,1\right)=x_{\text{P}}x_{\text{R}}^{\text{a}},\;Z\left(0,2\right)=x_{\text{R}}^{\text{a}}x_{\text{R}}^{\text{m}}. \end{array}$$ (14)

The procedure is analogous to adding up the weights indicated in the right column in Fig 2A.

Note that in general repressor molecules could bind to operator sites of two different gene copies at the same time, which has been observed in several experiments *in vitro* [52, 53]. It would be very interesting to study the effect of this situation on transcriptional regulation, especially in cases where the gene is located on mobile DNA elements such as plasmids. For the purposes of this paper, we restrict our attention to the simplest scenario and do not include those states in our partition function.

The fraction of promoters occupied by an RNAP molecule can by analogy to Eq (4), be calculated as

$$\begin{array}{c} \theta_{\text{P}}\left(\lambda_{\text{P}},\lambda_{\text{R}}\right)=\Xi^{-1}\left(\lambda_{\text{P}}x_{\text{P}}+\lambda_{\text{P}}\lambda_{\text{R}}x_{\text{P}}x_{\text{R}}^{\text{a}}\right). \end{array}$$ (15)

In the absence of repressors we have

$$\begin{array}{ccc} \theta_{\text{P}}\left(\lambda_{\text{P}},0\right) & = & \frac{\lambda_{\text{P}}x_{\text{P}}}{1+\lambda_{\text{P}}x_{\text{P}}}\\ & ≃ & \lambda_{\text{P}}x_{\text{P}}.\;\left(\lambda_{\text{P}}x_{\text{P}}\ll 1\right) \end{array}$$ (16)

The fold-change is given by the ratio of Eqs (15) and (16). Furthermore, we will again work in the weak promoter limit where *λ*_P_*x*_P_ ≪ 1, resulting in

$$\begin{array}{ccc} \text{fold-change} & = & \frac{\theta_{\text{P}}\left(\lambda_{\text{P}},\lambda_{\text{R}}\right)}{\theta_{\text{P}}\left(\lambda_{\text{P}},0\right)}\\ & ≃ & \frac{1+\lambda_{\text{R}}x_{\text{R}}^{\text{a}}}{1+\lambda_{\text{R}}\left(x_{\text{R}}^{\text{a}}+x_{\text{R}}^{\text{m}}+x_{\text{R}}^{\text{a}}x_{\text{R}}^{\text{m}}x_{\text{L}}\right)+\lambda_{\text{R}}^{2}x_{\text{R}}^{\text{m}}x_{\text{R}}^{\text{a}}}.\;\left(\lambda_{\text{P}}x_{\text{P}}\ll 1\right) \end{array}$$ (17)

**Fig 3:**
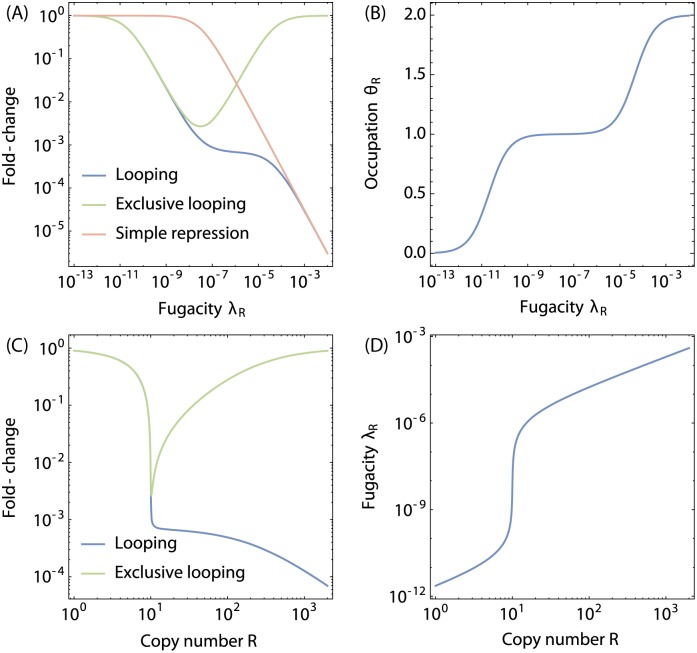
Fold-change and occupation for the looping scenarios. (**A**) Fold-change as a function of the fugacity *λ*_R_ for the looping scenario (blue curve, Eq (17)) and the exclusive looping scenario (green curve, Eq (20)). The pink curve is the simple repression scenario. (**B**) Average occupation of repressors to a single gene 〈*R*_ads_〉/*N* in Eq (22) in the looping architecture. (**C**) Fold-change as a function of the total number of repressor molecules *R* for the looping scenario (blue curve) and exclusive looping (green curve) scenario. (**D**) the repressor fugacity as function of the total number of repressor molecules *R* for both the looping and exclusive looping scenario. The value of $\epsilon_{\text{R}}^{m}=\epsilon_{\text{R}}^{a}=-17.3k_{\text{B}}T$, and *F*_L_ = +10*k*_B_*T* as in [34]. Furthermore, we took the number of promoters to be *N* = 10 and the number of non-specific sites to be *N*_ns_ = 5 × 10^6^.

The result of Eq (17) is shown in Fig 3. By comparing the result of Eq (17) with the canonical result as given in [45] (Eq 18.35 p. 827), we see that the two equations differ only in a substitution: We obtain the grand canonical result upon replacing in the canonical result *R*/*N*_ns_ by *λ*_R_ and $R\left(R-1\right)/N_{\text{ns}}^{2}$ by $\lambda_{\text{R}}^{2}$. We must stress that, except in the limit of *R* ≫ 1, the two ensembles are not equivalent—the value of *λ*_R_ is not equal to either *R*/*N*_ns_ or $\sqrt{R\left(R-1\right)/N_{\text{ns}}^{2}}$. The grand canonical fugacity *λ*_R_ merely plays the role of the canonical concentrations in otherwise identical expressions.

In order to facilitate a consistent comparison between theory and experimental gene activity data over different scenarios (here, simple repression and looping), we can write the result of Eq (17) in the same form as the simple repression result in Eq (9).

$$\begin{array}{c} \text{fold-change}=\frac{1}{1+z_{\text{L}}},\;\left(\text{looping}\right) \end{array}$$ (18)

where we have

$$\begin{array}{c} z_{\text{L}}=\frac{\lambda_{\text{R}}\left(x_{\text{R}}^{\text{m}}+x_{\text{R}}^{\text{a}}x_{\text{R}}^{\text{m}}x_{\text{L}}\right)+\lambda_{\text{R}}^{2}x_{\text{R}}^{\text{m}}x_{\text{R}}^{\text{a}}}{1+\lambda_{\text{R}}x_{\text{R}}^{\text{a}}}. \end{array}$$ (19)

This allows us to plot the experimentally determined fold-change of a promoter architecture against *z* = *λ*_R_*x*_R_ (for simple repression) or *z*_L_ (for the looping architecture), which should cause data from both types of promoter architecture to collapse onto the same scaling law (1 + *z*)^−1^.

### Exclusive looping

The exclusive looping architecture is a variant of the generic looping architecture where binding of RNAP to the promoter site is prohibited if and only if DNA looping occurs. For instance, a famous example of this is seen for AraC regulating the *araBAD* operon in the absence of arabinose [54]. In this case, RNAP is not prevented from binding next to a repressor occupied main operator. We will therefore have to consider two more configurations in addition to the ones shown in Fig 2A. These additional configurations are shown in Fig 2B together with their grand-canonical weights. Using the same procedure as in the previous section, we obtain the following expression for the fold-change in the exclusive looping scenario, written here in the same form as Eq (17) to allow a consistent comparison.

$$\begin{array}{c} \text{fold-change}=\frac{1}{1+z_{\text{EL}}},\;\left(\text{exclusive}\;\text{looping}\right) \end{array}$$ (20)

with the scaling factor *z*_EL_ given as

$$\begin{array}{c} z_{\text{EL}}=\frac{\lambda_{\text{R}}x_{\text{R}}^{\text{a}}x_{\text{R}}^{\text{m}}x_{\text{L}}}{1+\lambda_{\text{R}}\left(x_{\text{R}}^{\text{a}}+x_{\text{R}}^{\text{m}}\right)+\lambda_{\text{R}}^{2}x_{\text{R}}^{\text{a}}x_{\text{R}}^{\text{m}}}. \end{array}$$ (21)

Eq (20) is plotted in Fig 3A making it possible to compare the two different looping architectures. The consequence of exclusive looping is that repression is only effective at intermediate repressor concentrations. At lower fugacity, not enough repressor is present to cause repression, while at higher fugacities it becomes much more likely that both operators are occupied by two individual repressors, a situation that still allows RNAP to bind to the promoter.

### Finding the fugacity

We calculate the average number of adsorbed repressors onto both the main and auxiliary sites in the looping scenario illustrated in Fig 2A by

$$\begin{array}{ccc} \theta_{\text{R}}\left(\lambda_{\text{R}}\right) & = & \frac{\lambda_{\text{R}}}{\Xi }\frac{\partial \Xi }{\partial \lambda_{\text{R}}}\\ & = & \Xi^{-1}\left(\lambda_{\text{R}}\left(x_{\text{R}}^{\text{a}}+x_{\text{R}}^{\text{m}}+x_{\text{R}}^{\text{a}}x_{\text{R}}^{\text{m}}x_{\text{L}}\right)+2\lambda_{\text{R}}^{2}x_{\text{R}}^{\text{a}}x_{\text{R}}^{\text{m}}\right). \end{array}$$ (22)

The value of *θ*_R_ as a function of *λ*_R_ has been plotted in Fig 3B. As before, we work in the weak promoter limit (*λ*_P_*x*_P_ ≪ 1) and additionally, we set the average binding energy of the repressors to the *N*_ns_ non-specific binding sites to zero. The number of adsorbed repressors to non-specific sites in the situation that *λ*_R_ ≪ 1 (which is verified later) is given by

$$\begin{array}{c} \theta_{\text{R}}^{\text{ns}}=\frac{\lambda_{\text{R}}x_{\text{R}}^{\text{ns}}}{1+\lambda_{\text{R}}x_{\text{R}}^{\text{ns}}}≃\lambda_{\text{R}}. \end{array}$$ (23)

The value of *λ*_R_ follows by solving the mass balance equation for repressors

$$\begin{array}{c} R=N_{\text{ns}}\theta_{\text{R}}^{\text{ns}}+N\theta_{\text{R}}, \end{array}$$ (24)

which can be rewritten as a cubic equation of the form $a\lambda_{\text{R}}^{3}+b\lambda_{\text{R}}^{2}+c\lambda_{\text{R}}-R=0$, with coefficients *a*, *b* and *c* given by

$$\begin{array}{c} \begin{array}{c} a=x_{\text{R}}^{\text{a}}x_{\text{R}}^{\text{m}}N_{\text{ns}}\\ b=\left(x_{\text{R}}^{\text{a}}+x_{\text{R}}^{\text{m}}+x_{\text{R}}^{\text{a}}x_{\text{R}}^{\text{m}}x_{\text{L}}\right)N_{\text{ns}}+2x_{\text{R}}^{\text{a}}x_{\text{R}}^{\text{m}}N-x_{\text{R}}^{\text{a}}x_{\text{R}}^{\text{m}}R\\ c=N_{\text{ns}}+\left(x_{\text{R}}^{\text{a}}+x_{\text{R}}^{\text{m}}+x_{\text{R}}^{\text{a}}x_{\text{R}}^{\text{m}}x_{\text{L}}\right)\left(N-R\right). \end{array}\} \end{array}$$ (25)

The cubic equation has a positive real root

$$\begin{array}{c} \lambda_{\text{R}}=\Delta_{+}+\Delta_{-}-\frac{b}{3a}, \end{array}$$ (26)

with

$$\begin{array}{ccc} \Delta_{\pm } & = & {\left(C_{2}\pm \sqrt{C_{1}^{3}+C_{2}^{2}}\right)}^{1/3}\\ C_{1} & = & \left(c/3a\right)-{\left(b/3a\right)}^{2}\\ C_{2} & = & \left(bc/6a^{2}\right)+\left(R/2a\right)-{\left(b/3a\right)}^{3}. \end{array}$$ (27)

When different competing genes or other repressor binding sites are present, these can be included as an additional reservoir in Eq (24), at the cost of increasing the order of the polynomial to solve. In Fig 3(C) and 3(D) we plot fold-change and fugacity for the looping and exclusive looping scenario as a function of the number of repressors in the absence of competing genes.

The figures show several features, which can be explained by the degree of competition between the genes for the available number of transcription factors. The fugacity equals the reservoir concentration of transcription factors on the non-regulatory DNA. When the number of transcription factors is smaller than the number of genes, there is strong competition for the transcription factors. Consequently, the majority of the transcription factors are primarily adsorbed on the genes, while the non-regulatory reservoir is nearly empty. However, when the number of transcription factors exceeds the number of genes, the surplus of transcription factors reside in the non-regulatory reservoir, with a corresponding increase in fugacity. The crossover occurs when the number of transcription factors equals the number of genes. This leads to strong repression in both the looping and exclusive looping promoter architectures, since the most likely singly occupied configurational state for both architectures prohibits RNAP binding. When the concentration of transcription factors increases even more, the doubly occupied configurational states become more important which are repressive in the case of the looping architecture, but which allows transcription in the exclusive looping architectures.

### Looping and scaling

**Fig 4:**
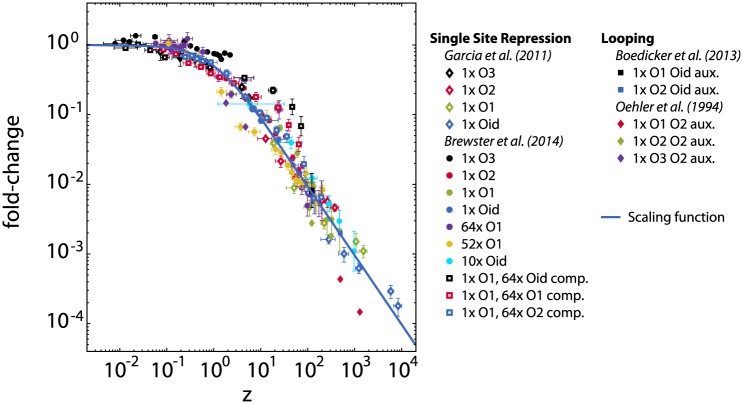
Transcription activity data of simple repression and looping regulated genes. Transcription activity data for the simple repression architecture from [17, 37], as previously shown in [38], as well as data for the looping scenario from [55, 56], rescaled to the scaling factor *z* appropriate to its architecture. For simple repression scenarios, *z* = *λ*_R_ exp(−*βϵ*_R_). For the looping scenario, *z*_L_ is calculated using Eqs (18) and (26). The solid blue line signifies the scaling function (1 + *z*)^−1^. The repressor binding energies are taken from [37] as $\epsilon_{\text{R}}^{\text{Oid}}=-17k_{\text{B}}T$, $\epsilon_{\text{R}}^{\text{O}1}=-15.3k_{\text{B}}T$,$\epsilon_{\text{R}}^{\text{O}2}=-13.9k_{\text{B}}T$ and $\epsilon_{\text{R}}^{\text{O}3}=-9.7k_{\text{B}}T$. Values for promoter copy numbers *N* and competitor sites *N*_c_ are taken from [17] (simple repression) and [55] (looping). The value for the looping free energy, *F*_L_ = +9.1*k*_B_*T*, was taken from Fig 3b in [55] as the average looping free energy for a loop that has a length in between 76 and 84 base pairs. For each data set, *λ*_R_ is calculated by solving the mass balance appropriate for the architecture, Eq (7) (simple repression) or Eq (24) (looping).

In Fig 4 we show available transcription data from the simple repression architecture. Data from [55, 56] were used to compare with the theory for looping architectures, in the form of the scaling function Eq (18), so that the results may be compared to the simple repression data. For the details see the caption of the Figure. It can be seen that when scaled in this form, the experimental data from the two repression architectures, that is, simple repression and looping, collapse to a single scaling function, as predicted in this and previous work [38]. The deviation from the curve of the data from [56] likely reflect the uncertainty in the number of repressors per cell reported, showing how sensitive this quantitative comparison is with respect to experimental uncertainties.

The fold-change of the simple repression and looping promoter architectures are dominated in the weak promoter limit by the occupation of the main operator site. Hence, when the expressions for fold-change are cast into the scaling form of Eq (18), the scaling parameter *z* could be interpreted as the relative weight of states where the main operator site is occupied, as modified by its surroundings. There is no deeper physical meaning that we attribute to the scaling parameter *z*. The exclusive looping architecture provides a borderline example that can still intuitively be mapped onto this functional form. The scaling parameter *z* here reflects the occupational weight of the looped state. However, there are many promoter architectures where the fold-change is not completely dominated by the occupation of a single main operator site, which limits the usability and interpretation of this scaling form in those cases.

## Activation

**Fig 5:**
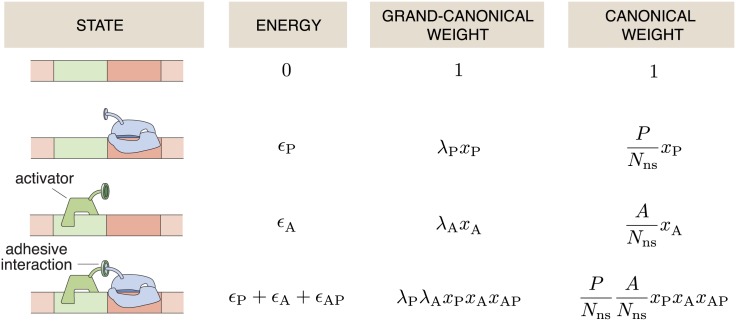
States and weights for the simple activation scenario. An activator and the RNA polymerase can bind to the activator binding site and to the promoter site with energies *ϵ*_A_ and *ϵ*_P_, respectively. The state where both molecules are bound simultaneously includes an additional energy *ϵ*_AP_, which reflects the adhesive interaction between activator and RNA polymerase.

In many situations, a transcription factor actively “recruits” RNAP to bind to a promoter. Essentially, there is an adhesive interaction between the bound transcription factor and the RNAP. In the following section we discuss the situation where genes are regulated by such an activator. The simplest of such situations, from now on referred to as simple activation, as well as the corresponding table of states and weights is shown in Fig 5.

An activator A and RNAP can bind to the operator site and promoter with energy *ϵ*_A_ and *ϵ*_P_, respectively. When both are bound to their appropriate sites simultaneously, there is an additional free energy gain of *ϵ*_AP_ which reflects the effective attraction between RNAP and activator. The situation in the canonical ensemble has been analyzed in [45](p. 810, Eq 19.6). That result will be compared to the fold-change expression which we derive below. We write the grand partition function for a single copy of an activator regulated gene as

$$\begin{array}{ccc} \Xi & = & \sum_{p=0}^{1}\sum_{a=0}^{1}\lambda_{\text{P}}^{p}\lambda_{\text{A}}^{a}Z\left(p,a\right)\\ & = & 1+\lambda_{\text{P}}x_{\text{P}}+\lambda_{\text{A}}x_{\text{A}}+\lambda_{\text{P}}\lambda_{\text{A}}x_{\text{P}}x_{\text{A}}x_{\text{AP}}, \end{array}$$ (28)

where $\lambda_{\text{A}}=e^{\beta \mu_{\text{A}}}$ is the fugacity of the activator with *μ*_A_ its chemical potential. Further we take *Z*(*p*, *a*) as: *Z*(0, 0) = 1, *Z*(1, 0) = *x*_P_, *Z*(0, 1) = *x*_A_, and *Z*(1, 1) = *x*_P_*x*_A_*x*_AP_. Here $x_{\text{A}}=e^{-\beta \epsilon_{\text{A}}}$ and $x_{\text{AP}}=e^{-\beta \epsilon_{\text{AP}}}$. The fraction of occupied promoter sites by RNAP is now given by

$$\begin{array}{c} \theta_{\text{P}}\left(\lambda_{\text{P}},\lambda_{\text{A}}\right)=\frac{\lambda_{\text{P}}x_{\text{P}}+\lambda_{\text{P}}\lambda_{\text{A}}x_{\text{P}}x_{\text{A}}x_{\text{AP}}}{1+\lambda_{\text{P}}x_{\text{P}}+\lambda_{\text{A}}x_{\text{A}}+\lambda_{\text{P}}\lambda_{\text{A}}x_{\text{P}}x_{\text{A}}x_{\text{AP}}}. \end{array}$$ (29)

In the absence of activators (*λ*_A_ = 0) we again regain Eq (5). Here we assumed that the fugacities of activator and RNAP, *λ*_P_, *λ*_A_ are independent, that is, *λ*_P_ has the same value in Eq (29) as it has in Eq (5), independent of the presence of activators. This is not trivial here, as activators interact with RNAP with energy *ϵ*_AP_. As shown in the supporting information (see S3 Text), decoupling is an excellent approximation as long as the number of non-specific sites is large. This is even the case when activators and RNAP can also have interactions with each other when both are bound to non-specific sites, which we also show in S2 Text. The fold-change is then found as

$$\begin{array}{c} \text{fold-change}=\frac{\theta_{\text{P}}\left(\lambda_{\text{P}},\lambda_{\text{A}}\right)}{\theta_{\text{P}}\left(\lambda_{\text{P}},0\right)}≃\frac{1+\lambda_{\text{A}}x_{\text{A}}x_{\text{AP}}}{1+\lambda_{\text{A}}x_{\text{A}}+\lambda_{\text{P}}\lambda_{\text{A}}x_{\text{P}}x_{\text{A}}x_{\text{AP}}}.\;\left(\lambda_{\text{P}}x_{\text{P}}\ll 1\right) \end{array}$$ (30)

In contrast to the simple repression and looping scenarios, the fold-change in the weak promoter limit is still dependent on the RNAP fugacity. Finding the fugacities therefore becomes a matter of solving a mass balance for activators and for RNAPs simultaneously. We can, however, greatly simplify the result if we assume that *λ*_P_*x*_P_*x*_AP_ ≪ 1. This is consistent with the weak promoter limit provided that *ϵ*_AP_ does not exceed several *k*_B_*T*. In that case, we can write

$$\begin{array}{c} \text{fold-change}=\frac{1+\lambda_{\text{A}}x_{\text{A}}x_{\text{AP}}}{1+\lambda_{\text{A}}x_{\text{A}}}.\;\left(\lambda_{\text{P}}x_{\text{P}}x_{\text{AP}}\ll 1\right) \end{array}$$ (31)

The fold-change expressions in the canonical ensemble for a single gene (Eq 19.6 p. 812 in [45]) and the grand canonical expression Eq (31) can again be related by replacing *A*/*N*_ns_ by *λ*_A_. Finding *λ*_A_ is analogous to the procedure described above for the looping scenarios.

## Comparison of canonical and grand-canonical fold-change expressions

**Fig 6:**
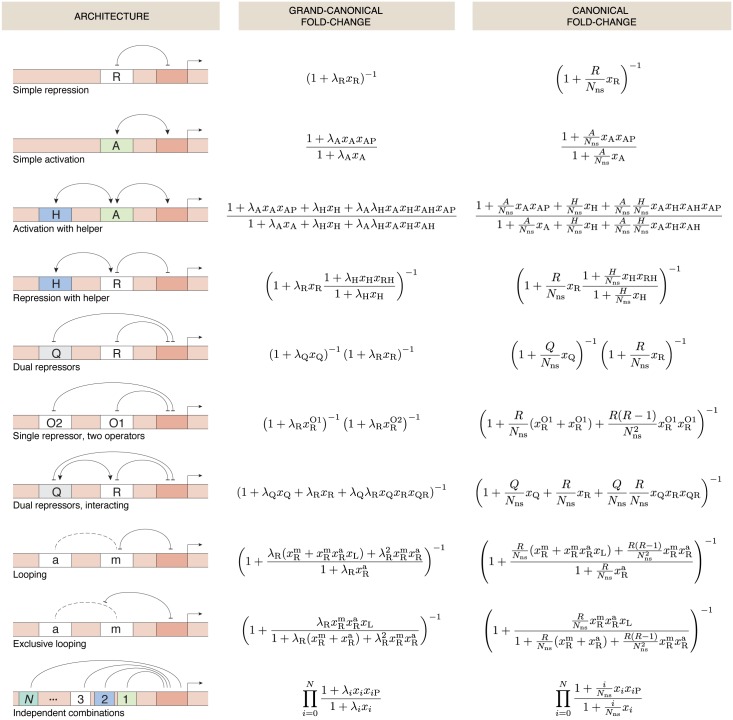
Fold-change in the grand canonical and the canonical ensemble for a variety of regulatory architectures. The promoter is indicated by a red patch on the DNA, with the transcription start site denoted by the straight arrow. Interactions between transcription factors bound to a site are specified by a solid curve ending in an arrow tip (activation), in a bar (repression) or unadorned (unspecified interaction). Dashed curved lines signify looping between two sites.

Fig 6 shows the fold-change expressions that were derived using the grand canonical ensemble for a variety of regulatory architectures, as well as the canonical expressions calculated in [7, 8]. The grand canonical expressions have the advantage that they analytically describe the situation where multiple genes or binding sites compete for transcription factors. In these competition scenarios, there can be multiple copies of the same gene or other genes that are regulated by the same transcription factors. The effect of competition is described by the transcription factor fugacity *λ*, which depends upon the nature (number, binding affinity) of additional binding reservoirs for that transcription factor.

The canonical expressions shown here, in contrast, describe only the case of an isolated, non-interacting gene. While the canonical ensemble can generally be used to describe the situation of multiple gene copies and competitor sites [17, 34], each competition scenario needs its own formula, which can be derived using combinatorics to explicitly account for all gene copies and competitor sites. Note, that each grand canonical expression for fold-change in Fig 6 differs from the canonical expression solely by a substitution of the concentration of the transcription factor by its fugacity, i.e. *R*/*N*_ns_ by *λ*_R_, *A*/*N*_ns_ by *λ*_A_ or $R\left(R-1\right)/N_{\text{ns}}^{2}$ by $\lambda_{\text{R}}^{2}$ respectively. The fugacity can be calculated for any competition scenario that consists of a finite number of competitor binding sites with known binding energies.

## Case study: The *lac* operon

**Fig 7:**
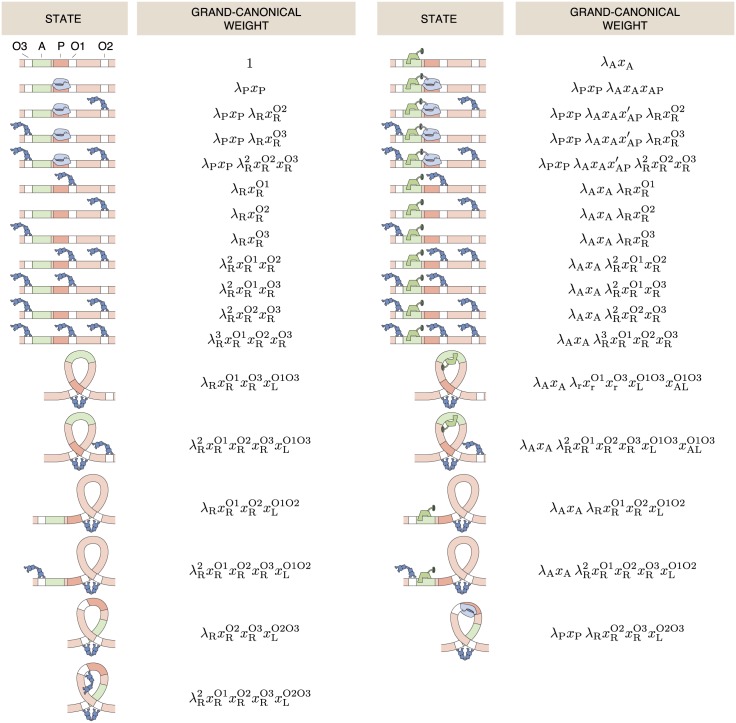
List of all allowed states of the *lac* operon, and their grand canonical weights. The *lac* operon has three binding sites (O1, O2, O3) for the *lac* repressor (LacI) and one binding (A) site for a CRP activator. LacI has two binding heads and can bind to two sites simultaneously. In those cases the DNA in between the binding sites forms a loop. States where RNAP is bound to the promoter (p) and LacI is bound to the O1 operator sites are not allowed, as well as looped states where RNAP is bound to the promoter.

In this case study we will show how to calculate the fold-change in gene expression for the regulatory motif of the *lac* operon in *Escherichia coli*. A sketch of the regulatory architecture is given in Fig 7. The architecture consists of a promoter site P next to an operator site O1 that binds the tetrameric repressor LacI, a protein that can bind 2 DNA sites simultaneously. There are two auxiliary operator sites O2 and O3, that also bind LacI, but binding of the repressor to these sites does not prevent the binding of RNAP to the promoter site. In this architecture, the repressor can bind to two operator sites at the same time which requires the DNA between the operator to form a loop. Furthermore, there is an activator site A that binds CRP, an activator which recruits RNAP for binding by making the adsorption of RNAP to the promoter site more favorable. Additionally, when the activator is bound to the adsorption site, the DNA is bent locally in such a way that the free energy penalty of a loop between the auxiliary repressor site O3 and the main site O1 is reduced [57–59].

### Grand partition function and fold-change

We write the grand partition function for the regulatory architecture of the *lac* operon as

$$\begin{array}{c} \Xi =\sum_{p=0}^{1}\sum_{r=0}^{3}\sum_{a=0}^{1}\lambda_{\text{P}}^{p}\lambda_{\text{R}}^{r}\lambda_{\text{A}}^{a}Z\left(p,r,a\right), \end{array}$$ (32)

where we have the fugacities $\lambda_{i}=e^{\beta \mu_{i}}$ as defined above and *Z*(*p*, *r*, *a*) the relevant part of the canonical partition function with *p*, *r*, *a* molecules of RNAP, LacI and CRP bound to the gene, respectively. Since the promoter sequence partly overlaps with the main operator site O1, their simultaneous occupation is prohibited by excluded volume interactions. Those states automatically have a weight of 0. There is a partial overlap between the activator site and the auxiliary operator site O3. LacI was found to bind to O3 even with CRP bound to the activator site, but its position is then shifted by 6 bp [58]. This, combined with the sharp bend in the DNA bound by CRP causes a change in the looping free energy of loop O1O3. For steric reasons, the loop O2O3 is thought not to occur when CRP is bound, so we have assigned those states a weight of 0. RNAP was found to bind to the promoter simultaneously with CRP while the auxiliary operator site O3 was occupied by LacI, but the favourable interaction between CRP and RNAP was decreased [16]. These states have been given the modified activator-RNAP interaction $\epsilon_{\text{AP}}^{\prime }$. We write out the sum by summing the weights of all the allowed occupational states noted in Fig 7 and obtain

$$\begin{array}{ccc} \Xi & = & \underset{\text{Free}\;\text{gene}}{\underset{︸}{1}}+\underset{\text{Activator}\;\text{bound}}{\underset{︸}{\lambda_{\text{A}}x_{\text{A}}}}\\ & & +\underset{\text{RNAP}\;\text{bound}\;\text{states}}{\underset{︸}{\lambda_{\text{P}}x_{\text{P}}\left\{\left(1+\lambda_{\text{A}}x_{\text{A}}x_{\text{AP}}\right)\left[1+\lambda_{\text{R}}\left(x_{\text{R}}^{\text{O}2}+x_{\text{R}}^{\text{O}3}\frac{x_{\text{AP}}^{\prime }}{x_{\text{AP}}}\right)+\lambda_{\text{R}}^{2}x_{\text{R}}^{\text{O}2}x_{\text{R}}^{\text{O}3}\frac{x_{\text{AP}}^{\prime }}{x_{\text{AP}}}\right]+\lambda_{\text{R}}x_{\text{R}}^{\text{O}2}x_{\text{R}}^{\text{O}3}x_{\text{L}}^{\text{O}2\text{O}3}\right\}}}\\ & & +\underset{\text{Only}\;\text{repressors}\;\text{bound,}\;\text{excluding}\;\text{looping}\;\text{states}}{\underset{︸}{\lambda_{\text{R}}\left(x_{\text{R}}^{\text{O}1}+x_{\text{R}}^{\text{O}2}+x_{\text{R}}^{\text{O}3}\right)+\lambda_{\text{R}}^{2}\left(x_{\text{R}}^{\text{O}1}x_{\text{R}}^{\text{O}2}+x_{\text{R}}^{\text{O}1}x_{\text{R}}^{\text{O}3}+x_{\text{R}}^{\text{O}2}x_{\text{R}}^{\text{O}3}\right)+\lambda_{\text{R}}^{3}x_{\text{R}}^{\text{O}1}x_{\text{R}}^{\text{O}2}x_{\text{R}}^{\text{O}3}}}\\ & & +\underset{\text{Activator}\;\text{and}\;\text{repressors}\;\text{bound,}\;\text{excluding}\;\text{looping}\;\text{states}}{\underset{︸}{\lambda_{\text{A}}x_{\text{A}}\left[\lambda_{\text{R}}\left(x_{\text{R}}^{\text{O}1}+x_{\text{R}}^{\text{O}2}+x_{\text{R}}^{\text{O}3}\right)+\lambda_{\text{R}}^{2}\left(x_{\text{R}}^{\text{O}1}x_{\text{R}}^{\text{O}2}+x_{\text{R}}^{\text{O}1}x_{\text{R}}^{\text{O}3}+x_{\text{R}}^{\text{O}2}x_{\text{R}}^{\text{O}3}\right)+\lambda_{\text{R}}^{3}x_{\text{R}}^{\text{O}1}x_{\text{R}}^{\text{O}2}x_{\text{R}}^{\text{O}3}\right]}}\\ & & +\underset{\text{Looping}\;\text{states}\;\text{with}\;\text{single}\;\text{repressor}}{\underset{︸}{\lambda_{\text{R}}\left(x_{\text{R}}^{\text{O}1}x_{\text{R}}^{\text{O}2}x_{\text{L}}^{\text{O}1\text{O}2}+x_{\text{R}}^{\text{O}1}x_{\text{R}}^{\text{O}3}x_{\text{L}}^{\text{O}1\text{O}3}+x_{\text{R}}^{\text{O}2}x_{\text{R}}^{\text{O}3}x_{\text{L}}^{\text{O}2\text{O}3}\right)}}\\ & & +\underset{\text{Looping}\;\text{states}\;\text{with}\;\text{single}\;\text{repressor,}\;\text{activator}\;\text{bound}}{\underset{︸}{\lambda_{\text{A}}x_{\text{A}}\;\lambda_{\text{R}}\left(x_{\text{R}}^{\text{O}1}x_{\text{R}}^{\text{O}2}x_{\text{L}}^{\text{O}1\text{O}2}+x_{\text{R}}^{\text{O}1}x_{\text{R}}^{\text{O}3}x_{\text{L}}^{\text{O}1\text{O}3}x_{\text{AL}}^{\text{O}1\text{O}3}\right)}}\\ & & +\underset{\text{Looping}\;\text{states}\;\text{with}\;\text{2}\;\text{repressors}\;\text{bound}}{\underset{︸}{\lambda_{\text{R}}^{2}x_{\text{R}}^{\text{O}1}x_{\text{R}}^{\text{O}2}x_{\text{R}}^{\text{O}3}\left(x_{\text{L}}^{\text{O}1\text{O}2}+x_{\text{L}}^{\text{O}1\text{O}3}+x_{\text{L}}^{\text{O}2\text{O}3}\right)}}\\ & & +\underset{\text{Looping}\;\text{states}\;\text{with}\;\text{2}\;\text{repressors}\;\text{bound,}\;\text{activator}\;\text{bound}}{\underset{︸}{\lambda_{\text{A}}x_{\text{A}}\;\lambda_{\text{R}}^{2}x_{\text{R}}^{\text{O}1}x_{\text{R}}^{\text{O}2}x_{\text{R}}^{\text{O}3}\left(x_{\text{L}}^{\text{O}1\text{O}2}+x_{\text{L}}^{\text{O}1\text{O}3}x_{\text{AL}}^{\text{O}1\text{O}3}\right)}}, \end{array}$$ (33)

where $x_{i}=e^{-\beta \epsilon_{i}}$ as before. Furthermore, *ϵ*_AP_ is the energy bonus that is gained by simultaneously binding RNAP and CRP, which when O3 is bound is modified to $\epsilon_{\text{AP}}^{\prime }$. Furthermore, $x_{\text{L}}^{ij}=\exp\left(-\beta F_{\text{L}}^{ij}\right)$ reflects the energy penalty needed to form a DNA loop between operators *i* and *j*, and $x_{\text{AL}}^{\text{O}1\text{O}3}=\exp\left(-\beta \Delta F_{\text{AL}}^{\text{O}1\text{O}3}\right)$ where $\Delta F_{\text{AL}}^{\text{O}1\text{O}3}$ is the change in looping free energy that results from simultaneously binding CRP and forming loop O1O3.

For simplicity of notation we split the grand partition function into terms that are linear with *λ*_P_*x*_P_ and those that are independent of *λ*_P_*x*_P_, so that we can write for the grand partition function

$$\begin{array}{c} \Xi =\lambda_{\text{P}}x_{\text{P}}\Sigma_{\text{P}}+\Sigma_{0}, \end{array}$$ (34)

where we have defined Σ_P_ as

$$\begin{array}{c} \Sigma_{\text{P}}\equiv \left(1+\lambda_{\text{A}}x_{\text{A}}x_{\text{AP}}\right)\left[1+\lambda_{\text{R}}\left(x_{\text{R}}^{\text{O}2}+x_{\text{R}}^{\text{O}3}\frac{x_{\text{AP}}^{\prime }}{x_{\text{AP}}}\right)+\lambda_{\text{R}}^{2}x_{\text{R}}^{\text{O}2}x_{\text{R}}^{\text{O}3}\frac{x_{\text{AP}}^{\prime }}{x_{\text{AP}}}\right]+\lambda_{\text{R}}x_{\text{R}}^{\text{O}2}x_{\text{R}}^{\text{O}3}x_{\text{L}}^{\text{O}2\text{O}3}, \end{array}$$ (35)

and with all states not leading to transcription initiation grouped as Σ_0_

$$\begin{array}{c} \Sigma_{0}\equiv \sum_{r=0}^{3}\sum_{a=0}^{1}\lambda_{\text{R}}^{r}\lambda_{\text{A}}^{A}Z\left(0,r,a\right). \end{array}$$ (36)

We then write the fraction of occupied promoter sites *θ*_P_ as

$$\begin{array}{c} \theta_{\text{P}}\left(\lambda_{\text{P}},\lambda_{\text{R}},\lambda_{\text{A}}\right)=\frac{\lambda_{\text{P}}}{\Xi }\frac{\partial \Xi }{\partial \lambda_{\text{P}}}=\frac{\lambda_{\text{P}}x_{\text{P}}\Sigma_{\text{P}}}{\lambda_{\text{P}}x_{\text{P}}\Sigma_{\text{P}}+\Sigma_{0}}. \end{array}$$ (37)

In the absence of any LacI or CRP the average number of occupied promoter sites *θ*_P_(*λ*_P_, 0, 0) is given as

$$\begin{array}{c} \theta_{\text{P}}\left(\lambda_{\text{P}},0,0\right)=\frac{\lambda_{\text{P}}x_{\text{P}}}{1+\lambda_{\text{P}}x_{\text{P}}}. \end{array}$$ (38)

As above, we can then find the fold-change as the ratio of the two. Thus, we write

$$\begin{array}{ccc} \text{fold-change} & = & \frac{\theta_{\text{P}}\left(\lambda_{\text{P}},\lambda_{\text{R}},\lambda_{\text{A}}\right)}{\theta_{\text{P}}\left(\lambda_{\text{P}},0,0\right)}=\frac{\left(1+\lambda_{\text{P}}x_{\text{P}}\right)\Sigma_{\text{P}}}{\lambda_{\text{P}}x_{\text{P}}\Sigma_{\text{P}}+\Sigma_{0}}\\ & ≃ & \frac{\Sigma_{\text{P}}}{\Sigma_{0}}.\;\left(\lambda_{\text{P}}x_{\text{P}}\ll 1,\;\lambda_{\text{P}}x_{\text{P}}\ll \frac{\Sigma_{0}}{\Sigma_{\text{P}}}\right) \end{array}$$ (39)

Here we have imposed the weak promoter limit *λ*_P_*x*_P_ ≪ 1, as well as a second assumption that *λ*_P_*x*_P_ ≪ Σ_0_/Σ_P_, which makes the fold-change independent of the RNAP fugacity. When repression is stronger than activation (which is the case when Σ_P_/Σ_0_ < 1), this second assumption is already implicit in the weak promoter limit. In the case of strong activation, however, this second assumption is stricter than the weak promoter limit and care needs to be taken when applying it. The validity of this assumption needs to evaluated a posteriori. As we will show in the supporting information (see S4 Text), this assumption is generally justified as long as the fold-change ≪ 500. If the assumption breaks down, the RNAP fugacity *λ*_P_ needs to be calculated explicitly.

### Imposing the constraint of fixed transcription factor numbers

The fugacities *λ*_R_ and *λ*_A_ can be found self-consistently by imposing the constraint that the total number of repressors *R* and activators *A* in the cell is conserved. We set up two mass balances which we will then decouple. LacI is not shared with other genes in the cell, hence our choice not to include any competing reservoir for LacI. In contrast, CRP binds to approximately 350 other sites [49]. We therefore include an additional reservoir of competing sites for CRP, reflecting the high degree to which CRP is shared between genes.

#### Activators

For the conservation of CRP, we can write down the following mass balance

$$\begin{array}{c} A=N_{\text{ns}}\theta_{\text{A}}^{\text{ns}}+N_{\text{c}}\theta_{\text{A}}^{\text{c}}+N\theta_{\text{A}}. \end{array}$$ (40)

Here, we have *N*_ns_ non-specific sites, *N* specific sites and *N*_c_ competitor sites. Each reservoir has its own occupation fraction. The fraction of CRP bound non-specific sites can be found as above as

$$\begin{array}{c} \theta_{\text{A}}^{\text{ns}}=\frac{\lambda_{\text{A}}x_{\text{A}}^{\text{ns}}}{1+\lambda_{\text{A}}x_{\text{A}}^{\text{ns}}}≃\lambda_{\text{A}}.\;\left(\lambda_{\text{A}}\ll 1\right) \end{array}$$ (41)

As before, we have set the reference point of energy to the binding energy of non-specific sites, hence $x_{\text{A}}^{\text{ns}}=e^{0}=1$. We assume the competitor sites to be sites to which CRP can bind with a binding energy $\epsilon_{\text{A}}^{\text{c}}$. The fraction of occupied competitor sites is then found as

$$\begin{array}{c} \theta_{\text{A}}^{\text{c}}=\frac{\lambda_{\text{A}}x_{\text{A}}^{\text{c}}}{1+\lambda_{\text{A}}x_{\text{A}}^{\text{c}}}. \end{array}$$ (42)

The fraction of CRP bound specific sites is calculated as

$$\begin{array}{c} \theta_{\text{A}}=\frac{\lambda_{\text{A}}}{\Xi }\frac{\partial \Xi }{\partial \lambda_{\text{A}}}≃\frac{\lambda_{\text{A}}}{\Sigma_{0}}\frac{\partial \Sigma_{0}}{\partial \lambda_{\text{A}}},\;\left(\lambda_{\text{P}}x_{\text{P}}\ll \Sigma_{0}/\Sigma_{\text{P}}\right) \end{array}$$ (43)

$$\begin{array}{c} =\frac{\lambda_{\text{A}}x_{\text{A}}f}{1+\lambda_{\text{A}}x_{\text{A}}f}. \end{array}$$ (44)

Here, we have simplified this expression by neglecting all the terms that are linear in *λ*_P_*x*_P_, provided that *λ*_P_*x*_P_ ≪ Σ_0_/Σ_P_, and we have grouped all the *λ*_R_-dependent terms in the factor *f*. Essentially, *λ*_A_*f* now behaves as an effective concentration in a Langmuir-like adsorption isotherm, where the effect of repressors is isolated in the factor *f*, given by

$$\begin{array}{ccc} f & = & [1+\lambda_{\text{R}}\left(x_{\text{R}}^{\text{O}1}+x_{\text{R}}^{\text{O}2}+x_{\text{R}}^{\text{O}3}\right)+\lambda_{\text{R}}^{2}\left(x_{\text{R}}^{\text{O}1}x_{\text{R}}^{\text{O}2}+x_{\text{R}}^{\text{O}1}x_{\text{R}}^{\text{O}3}+x_{\text{R}}^{\text{O}2}x_{\text{R}}^{\text{O}3}\right)+\lambda_{\text{R}}^{3}x_{\text{R}}^{\text{O}1}x_{\text{R}}^{\text{O}2}x_{\text{R}}^{\text{O}3}\\ & & +\lambda_{\text{R}}\left(x_{\text{R}}^{\text{O}1}x_{\text{R}}^{\text{O}2}x_{\text{L}}^{\text{O}1\text{O}2}+x_{\text{R}}^{\text{O}1}x_{\text{R}}^{\text{O}3}x_{\text{L}}^{\text{O}1\text{O}3}x_{\text{AL}}^{\text{O}1\text{O}3}\right)+\lambda_{\text{R}}^{2}x_{\text{R}}^{\text{O}1}x_{\text{R}}^{\text{O}2}x_{\text{R}}^{\text{O}3}\left(x_{\text{L}}^{\text{O}1\text{O}2}+x_{\text{L}}^{\text{O}1\text{O}3}x_{\text{AL}}^{\text{O}1\text{O}3}\right)]\\ & & \times [1+\lambda_{\text{R}}\left(x_{\text{R}}^{\text{O}1}+x_{\text{R}}^{\text{O}2}+x_{\text{R}}^{\text{O}3}\right)+\lambda_{\text{R}}^{2}\left(x_{\text{R}}^{\text{O}1}x_{\text{R}}^{\text{O}2}+x_{\text{R}}^{\text{O}1}x_{\text{R}}^{\text{O}3}+x_{\text{R}}^{\text{O}2}x_{\text{R}}^{\text{O}3}\right)+\lambda_{\text{R}}^{3}x_{\text{R}}^{\text{O}1}x_{\text{R}}^{\text{O}2}x_{\text{R}}^{\text{O}3}\\ & & +\lambda_{\text{R}}\left(x_{\text{R}}^{\text{O}1}x_{\text{R}}^{\text{O}2}x_{\text{L}}^{\text{O}1\text{O}2}+x_{\text{R}}^{\text{O}1}x_{\text{R}}^{\text{O}3}x_{\text{L}}^{\text{O}1\text{O}3}+x_{\text{R}}^{\text{O}2}x_{\text{R}}^{\text{O}3}x_{\text{L}}^{\text{O}2\text{O}3}\right)\\ & & +{\lambda_{\text{R}}^{2}x_{\text{R}}^{\text{O}1}x_{\text{R}}^{\text{O}2}x_{\text{R}}^{\text{O}3}\left(x_{\text{L}}^{\text{O}1\text{O}2}+x_{\text{L}}^{\text{O}1\text{O}3}+x_{\text{L}}^{\text{O}2\text{O}3}\right)]}^{-1}. \end{array}$$ (45)

Setting up the mass balance in Eq (40) leads to a cubic equation (in the absence of competitor sites, this reduces to a quadratic equation) that can be solved analytically.

$$\begin{array}{c} a\lambda_{\text{A}}^{3}+b\lambda_{\text{A}}^{2}+c\lambda_{\text{A}}-A=0, \end{array}$$ (46)

with coefficients

$$\begin{array}{ccc} a & = & N_{\text{ns}}x_{\text{A}}x_{\text{A}}^{\text{c}}f\\ b & = & N_{\text{ns}}\left(x_{\text{A}}f+x_{\text{A}}^{\text{c}}\right)+\left(N+N_{\text{c}}-A\right)x_{\text{A}}x_{\text{A}}^{\text{c}}f\\ c & = & N_{\text{ns}}+\left(N-A\right)x_{\text{A}}f+\left(N_{\text{c}}-A\right)x_{\text{A}}^{\text{c}}. \end{array}$$ (47)

Its solution remains a function of the repressor fugacity, however. The positive real root of the cubic equation is given by

$$\begin{array}{c} \lambda_{\text{A}}=\Delta_{+}+\Delta_{-}-\frac{b}{3a}, \end{array}$$ (48)

with

$$\begin{array}{ccc} \Delta_{\pm } & = & \sqrt[{3}]{C_{2}\pm \sqrt{C_{1}^{3}+C_{2}^{2}}}\\ C_{1} & = & \left(c/3a\right)-{\left(b/3a\right)}^{2}\\ C_{2} & = & \left(bc/6a^{2}\right)+\left(A/2a\right)-{\left(b/3a\right)}^{3}. \end{array}$$ (49)

#### Repressors

In order to determine the repressor fugacity *λ*_R_, we write down the mass balance of repressor molecules in the absence of additional reservoirs as

$$\begin{array}{c} R=N_{\text{ns}}\theta_{\text{R}}^{\text{ns}}+N\theta_{\text{R}}, \end{array}$$ (50)

where the average number of repressors bound to a non-specific site is, as in the case of activators (see Eq (41)), $\theta_{\text{R}}^{\text{ns}}≃\lambda_{\text{R}}$. The average number of repressors bound to a gene is, as before, given by

$$\begin{array}{c} \theta_{\text{R}}=\frac{\lambda_{\text{R}}}{\Xi }\frac{\partial \Xi }{\partial \lambda_{\text{R}}}≃\frac{\lambda_{\text{R}}}{\Sigma_{0}}\frac{\partial \Sigma_{0}}{\partial \lambda_{\text{R}}},\;\left(\lambda_{\text{P}}x_{\text{P}}\ll \Sigma_{0}/\Sigma_{\text{P}}\right). \end{array}$$ (51)

As before, we simplify this result in the weak promoter limit by neglecting the terms that are linear with *λ*_P_*x*_P_, which is a good approximation provided that *λ*_P_*x*_P_ ≪ Σ_0_/Σ_P_. This also resolves any indirect coupling that *λ*_R_ and *λ*_A_ have via their mutual interaction with *λ*_P_. The fugacities *λ*_R_ and *λ*_A_ are, however, still coupled through their direct interaction. Writing out Eq (51) explicitly, we obtain

$$\begin{array}{ccc} \theta_{\text{R}} & = & [\lambda_{\text{R}}\left(x_{\text{R}}^{\text{O}1}+x_{\text{R}}^{\text{O}2}+x_{\text{R}}^{\text{O}3}\right)+2\lambda_{\text{R}}^{2}\left(x_{\text{R}}^{\text{O}1}x_{\text{R}}^{\text{O}2}+x_{\text{R}}^{\text{O}1}x_{\text{R}}^{\text{O}3}+x_{\text{R}}^{\text{O}2}x_{\text{R}}^{\text{O}3}\right)\\ & & +3\lambda_{\text{R}}^{3}x_{\text{R}}^{\text{O}1}x_{\text{R}}^{\text{O}2}x_{\text{R}}^{\text{O}3}+\lambda_{\text{R}}\left(x_{\text{R}}^{\text{O}1}x_{\text{R}}^{\text{O}2}x_{\text{L}}^{\text{O}1\text{O}2}+x_{\text{R}}^{\text{O}1}x_{\text{R}}^{\text{O}3}x_{\text{L}}^{\text{O}1\text{O}3}g+x_{\text{R}}^{\text{O}2}x_{\text{R}}^{\text{O}3}x_{\text{L}}^{\text{O}2\text{O}3}h\right)\\ & & +2\lambda_{\text{R}}^{2}x_{\text{R}}^{\text{O}1}x_{\text{R}}^{\text{O}2}x_{\text{R}}^{\text{O}3}\left(x_{\text{L}}^{\text{O}1\text{O}2}+x_{\text{L}}^{\text{O}1\text{O}3}g+x_{\text{L}}^{\text{O}2\text{O}3}h\right)]\\ & & \times [1+\lambda_{\text{R}}\left(x_{\text{R}}^{\text{O}1}+x_{\text{R}}^{\text{O}2}+x_{\text{R}}^{\text{O}3}\right)+\lambda_{\text{R}}^{2}\left(x_{\text{R}}^{\text{O}1}x_{\text{R}}^{\text{O}2}+x_{\text{R}}^{\text{O}1}x_{\text{R}}^{\text{O}3}+x_{\text{R}}^{\text{O}2}x_{\text{R}}^{\text{O}3}\right)\\ & & +\lambda_{\text{R}}^{3}x_{\text{R}}^{\text{O}1}x_{\text{R}}^{\text{O}2}x_{\text{R}}^{\text{O}3}+\lambda_{\text{R}}\left(x_{\text{R}}^{\text{O}1}x_{\text{R}}^{\text{O}2}x_{\text{L}}^{\text{O}1\text{O}2}+x_{\text{R}}^{\text{O}1}x_{\text{R}}^{\text{O}3}x_{\text{L}}^{\text{O}1\text{O}3}g+x_{\text{R}}^{\text{O}2}x_{\text{R}}^{\text{O}3}x_{\text{L}}^{\text{O}2\text{O}3}h\right)\\ & & +{\lambda_{\text{R}}^{2}x_{\text{R}}^{\text{O}1}x_{\text{R}}^{\text{O}2}x_{\text{R}}^{\text{O}3}\left(x_{\text{L}}^{\text{O}1\text{O}2}+x_{\text{L}}^{\text{O}1\text{O}3}g+x_{\text{L}}^{\text{O}2\text{O}3}h\right)]}^{-1}, \end{array}$$ (52)

where we have isolated the *λ*_A_-dependent terms into the factors *g* and *h* given by

$$\begin{array}{c} g\equiv \frac{1+\lambda_{\text{A}}x_{\text{A}}x_{\text{AL}}^{\text{O}1\text{O}3}}{1+\lambda_{\text{A}}x_{\text{A}}},\;h\equiv \frac{1}{1+\lambda_{\text{A}}x_{\text{A}}}. \end{array}$$ (53)

Eq (50) leads to a quartic equation in *λ*_R_ of the form

$$\begin{array}{c} a\lambda_{\text{R}}^{4}+b\lambda_{\text{R}}^{3}+c\lambda_{\text{R}}^{2}+d\lambda_{\text{R}}-R=0, \end{array}$$ (54)

with the coefficients given by

$$\begin{array}{ccc} a & = & x_{\text{R}}^{\text{O}1}x_{\text{R}}^{\text{O}2}x_{\text{R}}^{\text{O}3}N_{\text{ns}}\\ b & = & \left\{ \begin{array}{c} x_{\text{R}}^{\text{O}1}x_{\text{R}}^{\text{O}2}x_{\text{R}}^{\text{O}3}\left(3N-R\right)+[x_{\text{R}}^{\text{O}1}x_{\text{R}}^{\text{O}2}+x_{\text{R}}^{\text{O}1}x_{\text{R}}^{\text{O}3}+x_{\text{R}}^{\text{O}2}x_{\text{R}}^{\text{O}3}\\ +x_{\text{R}}^{\text{O}1}x_{\text{R}}^{\text{O}2}x_{\text{R}}^{\text{O}3}\left(x_{\text{L}}^{\text{O}1\text{O}2}+x_{\text{L}}^{\text{O}1\text{O}3}g+x_{\text{L}}^{\text{O}2\text{O}3}h\right)]N_{\text{ns}} \end{array}\right.\\ c & = & \left\{ \begin{array}{c} (x_{\text{R}}^{\text{O}1}+x_{\text{R}}^{\text{O}2}+x_{\text{R}}^{\text{O}3}+x_{\text{R}}^{\text{O}1}x_{\text{R}}^{\text{O}2}x_{\text{L}}^{\text{O}1\text{O}2}+x_{\text{R}}^{\text{O}1}x_{\text{R}}^{\text{O}3}x_{\text{L}}^{\text{O}1\text{O}3}g\\ +x_{\text{R}}^{\text{O}2}x_{\text{R}}^{\text{O}3}x_{\text{L}}^{\text{O}2\text{O}3}h)N_{\text{ns}}+[x_{\text{R}}^{\text{O}1}x_{\text{R}}^{\text{O}2}+x_{\text{R}}^{\text{O}1}x_{\text{R}}^{\text{O}3}+x_{\text{R}}^{\text{O}2}x_{\text{R}}^{\text{O}3}\\ +x_{\text{R}}^{\text{O}1}x_{\text{R}}^{\text{O}2}x_{\text{R}}^{\text{O}3}\left(x_{\text{L}}^{\text{O}1\text{O}2}+x_{\text{L}}^{\text{O}1\text{O}3}g+x_{\text{L}}^{\text{O}2\text{O}3}h\right)]\left(2N-R\right) \end{array}\right.\\ d & = & \left\{ \begin{array}{c} (x_{\text{R}}^{\text{O}1}+x_{\text{R}}^{\text{O}2}+x_{\text{R}}^{\text{O}3}+x_{\text{R}}^{\text{O}1}x_{\text{R}}^{\text{O}2}x_{\text{L}}^{\text{O}1\text{O}2}+x_{\text{R}}^{\text{O}1}x_{\text{R}}^{\text{O}3}x_{\text{L}}^{\text{O}1\text{O}3}g\\ +x_{\text{R}}^{\text{O}2}x_{\text{R}}^{\text{O}3}x_{\text{L}}^{\text{O}2\text{O}3}h)\left(N-R\right)+N_{\text{ns}}. \end{array}\right. \end{array}$$ (55)

The quartic equation has four analytical roots, of which the positive real one is the desired solution, given by

$$\begin{array}{ccc} \lambda_{\text{R}} & = & -\frac{b}{4a}+\frac{1}{2}\sqrt{\frac{b^{2}}{4a^{2}}-\frac{2c}{3a}+\frac{\Delta_{0}}{3Q}+\frac{Q}{3}}\\ & & +\frac{1}{2}\sqrt{\frac{b^{2}}{2a^{2}}-\frac{4c}{3a}-\frac{\Delta_{0}}{3Q}-\frac{Q}{3}+\frac{-b^{3}a^{-3}+4bca^{-2}-8da^{-1}}{4\sqrt{\frac{b^{2}}{4a^{2}}-\frac{2c}{3a}+\frac{\Delta_{0}}{3Q}+\frac{Q}{3}}}}, \end{array}$$ (56)

with

$$\begin{array}{ccc} Q & = & \sqrt[{3}]{\frac{\Delta_{1}+\sqrt{-4\Delta_{1}^{3}+\Delta_{0}^{2}}}{2}}\\ \Delta_{0} & = & \frac{c^{2}}{a^{2}}-\frac{3bd}{a^{2}}-\frac{12R}{a}\\ \Delta_{1} & = & \frac{2c^{3}}{a^{3}}-\frac{9bcd}{a^{3}}+\frac{27d^{2}}{a^{2}}-\frac{27b^{2}R}{a^{3}}+\frac{72cR}{a^{2}}. \end{array}$$ (57)

**Fig 8:**
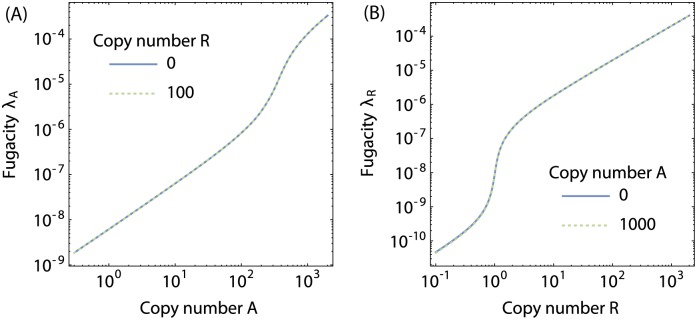
Fugacities of the transcription factors for the *lac* operon. (**A**) Fugacity of activators as a function of the number of activator molecules in the cell, in the absence of repressor (blue curve) and in the presence of a high concentration of repressor (green dotted curve). (**B**) Fugacity of repressors as a function of the number of repressors in the cell, in the absence of activator (blue curve) and in the presence of a high concentration of activator (green dotted curve). In both cases the copy number of the gene is *N* = 1. Note that the presence of repressor causes a slight shift in the activator fugacity. The parameters used are listed in Table 2.

Fig 8 shows the fugacities *λ*_A_ and *λ*_R_ as a function of transcription factor copy number in the absence and presence of the coupled complementary transcription factor. It can be seen that the difference between the unperturbed (i.e. in the absence of the complementary transcription factor) and the perturbed fugacities is negligible, and consequently it makes sense to decouple the activator and repressor fugacities completely (*f* = *g* = *h* = 1). We show in S1 Text Text how to decouple the transcription factor fugacities in the case that the perturbed fugacity deviates from the unperturbed fugacity.

The fugacities *λ*_A_ and *λ*_R_, shown in Fig 8, both show similar features. At high transcription factor copy number there is a surplus of transcription factors, and the transcription factors are not strongly competed for. When only a handful of LacI repressors are present in the cell, the favourable binding of LacI to its cognate operator sites causes the operon to compete strongly for the few available LacI molecules. In turn, this causes a sharp decrease in the reservoir concentration, hence the crossover in fugacity when the number of LacI repressors approximately matches the gene copy number. In contrast, CRP is strongly competed for by approximately 350 other genes and consequently the crossover from high competition to transcription factor surplus occurs at CRP copy numbers between 10^2^ and 10^3^.

### Results and discussion

Most of the adsorption and interaction energies that are relevant to our calculations are known from previous experiments; only the looping free energies of the lesser studied O1O2 and O2O3 loops, the coupling strength between activator and the O1O3 loop and the reduction in the activation when O3 is occupied have yet to be verified by independent experimental studies. In general, the looping free energy depends on several factors, notably the length of the loop and the number of stable conformations that can be formed in conjunction with the tetrameric repressor. These interactions can be modeled explicitly as is done in e.g. refs [60, 61]. Table 2 shows the experimentally determined values of the different adsorption and looping free energies that are known [45].

**Table 2:** Physical absorption and interaction energies used. All data is obtained from [45], unless stated otherwise.

Symbol	Energy / *k*_B_*T*	Notes
$\epsilon_{\text{R}}^{\text{O}1}$	-15.3	
$\epsilon_{\text{R}}^{\text{O}2}$	-13.9	
$\epsilon_{\text{R}}^{\text{O}3}$	-9.7	
$F_{\text{L}}^{\text{O}2\text{O}3}$	>5	*
$F_{\text{L}}^{\text{O}1\text{O}3}$	9.1	
$F_{\text{L}}^{\text{O}1\text{O}2}$	7.6	^†^
*ϵ*_A_	-13.0	[63]
*ϵ*_AP_	-5.3	[63]
$\epsilon_{\text{AP}}^{\prime }$	-1.8	^§^
$\Delta F_{\text{AL}}^{\text{O}1\text{O}3}$	-3.4	^‡^

*This loop does not occur in the presence of CRP and could not be calibrated to the data available in [56]

^†^From calibration to [56] from construct with deleted O3 auxiliary site.

^‡^From calibration to [56] from construct with deleted O2 auxiliary site.

^§^From calibration to [56] from constructs with deleted main and O2 auxiliary site.

We calibrated the model to existing experiments on the *lac* operon to find the missing energies. In a range of experiments by Oehler in the 1990’s [56, 62], the fold-change of the *lac* operon was determined in the presence of two concentrations of *lac* repressor. Different constructs were tested, where some adsorption sites were deleted from the genome, or replaced by the sequence of a different operator. While there exists an uncertainty in the actual number of repressors in these experiments, the number of different mutations that were tested make this study a prime candidate to calibrate the model. Note also that in these experiments the activator site was kept intact, but Oehler *et al.* did not actively control the number of activators, nor report their concentration. We have assumed a number of ∼1000 activators [45], at which the activator sites are more or less saturated. Furthermore, we have used a total of *N*_c_ = 350 competitor sites [49], each with an estimated binding energy for CRP of $\epsilon_{\text{A}}^{\text{c}}=-13k_{\text{B}}T$.

We found *R*, $F_{\text{L}}^{\text{O}1\text{O}2}$, $\Delta F_{\text{AL}}^{\text{O}1\text{O}3}$, and $\epsilon_{\text{AP}}^{\prime }$ by calibrating the model to the constructs with deleted O2 and O3 sites, deleted O3 site, deleted O2 site, and deleted O1 and O2 respectively. In the presence of physiological numbers of CRP, the loop O2O3 is almost completely suppressed. With no experimental data in the absence of CRP, the looping free energy $F_{\text{L}}^{\text{O}2\text{O}3}$ could not be determined accurately.

**Fig 9:**
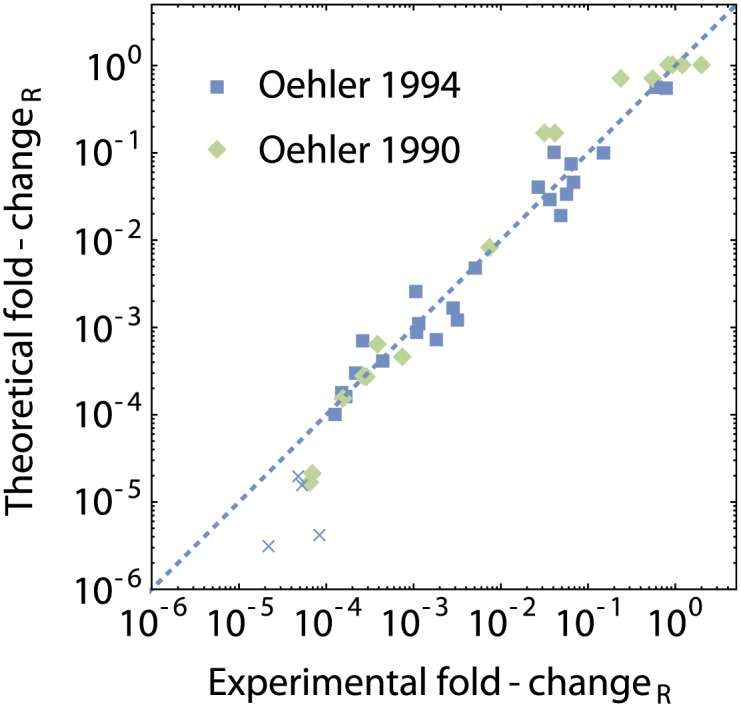
Fold-change of *lac* operon constructs from the literature. Theoretical fold-change according to Eq (39) compared to the experimental fold-change as determined in [56, 62]. The dashed line is the *x* = *y* line. The blue squares correspond to the 1994 paper, the green diamonds to the older 1990 paper. For some strongly repressive constructs, Oehler *et al.* were only able to measure a lower bound to the level of repression. These points were marked with a cross.

We plot the results of Oehler *et al.* in Fig 9, after calibration of the model. The experimentally determined fold-change (normalized in the presence of CRP) was plotted on the *x*-axis of the graph, and the corresponding theoretical fold-change on the *y*-axis, with perfect correspondence between experiment and theory when a point falls on the *x* = *y* line that is shown as the blue dotted line in the graph. Most points in the classical results of Oehler *et al.* fall within five-fold of perfect correspondence, over a very wide range of experimental parameters. For some very repressive constructs, Oehler *et al.* were only able to determine a lower bound to the level of repression (defined there as the reciprocal of the fold-change). Those constructs have been marked with a cross in Fig 9. Those points all fall right of the *x* = *y* line, indicating that the theoretical framework indeed predicts a lower activity than could be seen experimentally.

Vilar and Saiz [16] have proposed a model of the *lac* operon based on the canonical ensemble, which also captures the behaviour of the classical experiments by Oehler *et al.* In their canonical framework they have included explicitly the association equilibrium of LacI dimers to tetramers, and the binding of LacI to external inducer. Their model appears to be similar in accuracy to ours. Their use of the canonical ensemble is justified here since they do not introduce the CRP activators explicitly. Rather, they scale the effect of reduced activation by the occupation of the O3 auxiliary site with an effective fit parameter. Since LacI is not strongly competed for in wildtype cells, there is no similar titration effect such as is the case for CRP. When CRP is modeled explicitly, or when LacI is competed for, for example by competitor genes or competitive inhibitors, the titration effect that arises needs to be dealt with, and those situations can be modeled in the grand canonical framework. Moreover, the association equilibrium of LacI dimers to tetramers, and the binding of LacI by inducers can be introduced into the framework in a straightforward way.

**Fig 10:**
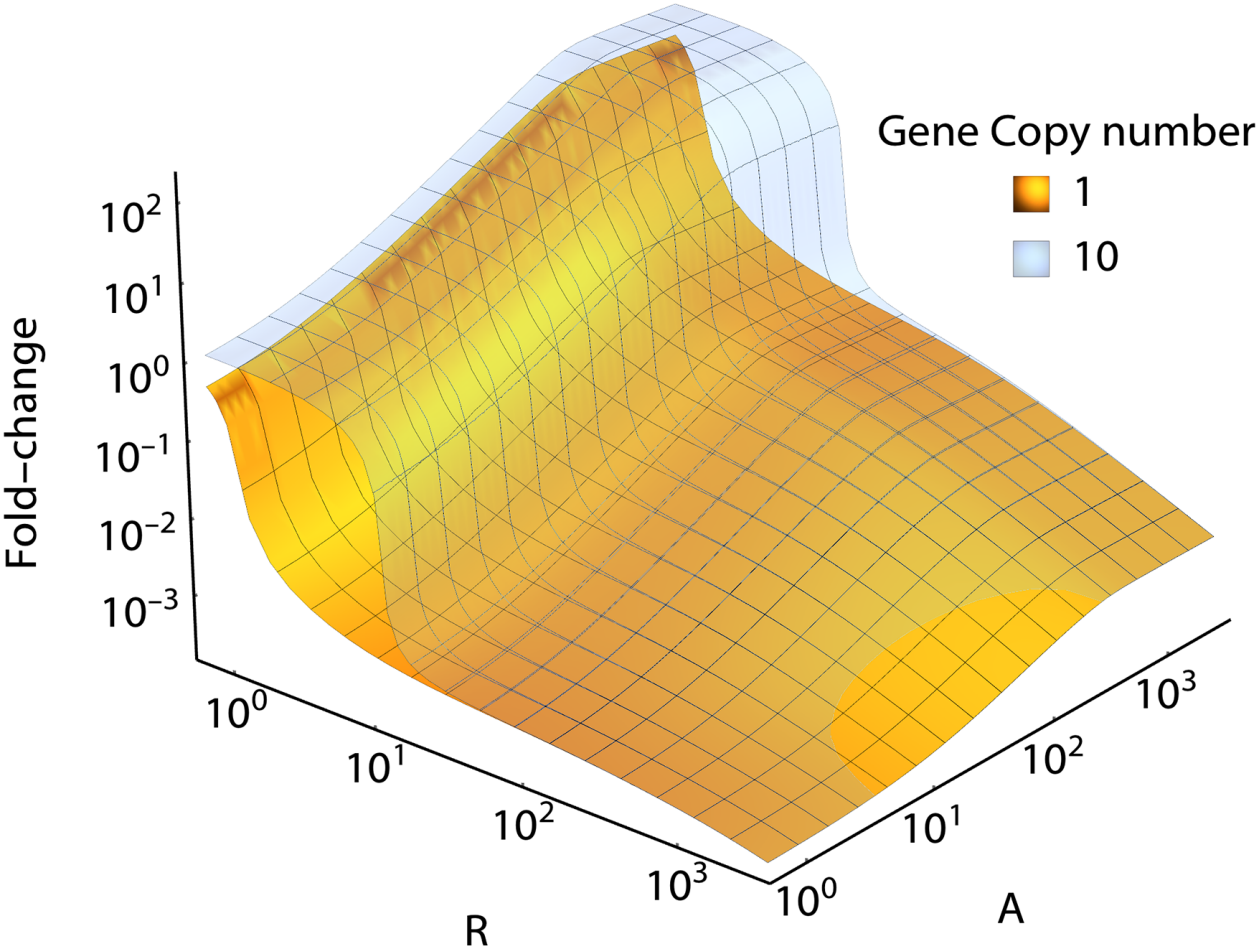
Fold-change of the *lac* operon. Fold-change as a function of activator and repressor concentrations for *N* = 1 (yellow surface) and *N* = 10 (translucent blue surface). When only a single copy of the *lac* operon is present in the cell, the action of LacI is significant: the introduction of as little as 2 or 3 copies of LacI cause a 100-fold drop in the transcription rate. In vivo, *E. coli* cells typically contain 10^1^ instances of LacI, keeping the activity of the *lac* operon low. When there are multiple copies of the *lac* operon present, all copies have to compete for the availability of LacI and significant repression only occurs when the number of LacI exceeds the operon copy number. Due to this titration effect, the transcription rate becomes sensitive to fluctuations in wildtype LacI availability. A similar titration effect occurs for the availability of CRP, but since CRP is already strongly competed for, the addition of multiple gene copies has no significant additional effect.

Fig 10 shows the cooperative effect of activators and repressors on the fold-change of the operon. As expected, addition of activators leads to an increase in the fold-change at low repressor copy number.

**Fig 11:**
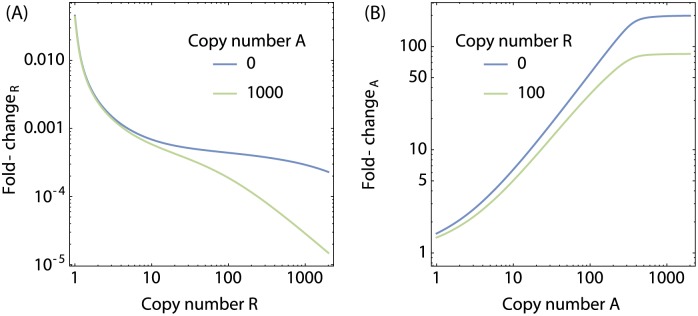
Activators increase the dynamic range of repression of the *lac* operon. (**A**) Gene expression normalized to the gene expression at *R* = 0 (fold-change_R_) as a function of number of repressors, in the absence (blue curve) and presence (green) of activators. (**B**) Gene expression normalized to the gene expression at *A* = 0 (fold-change_A_) as a function of the number of activators, in the absence (blue curve) and presence (green curve) of repressors. Bound activator causes a sharp bend in the DNA that facilitates the loop between O1 and O3. This causes an additional, cooperative repression effect on top of the (uncooperative) activation behaviour of the activators.

However, an interesting cooperative effect occurs in the presence of CRP. The presence of activators increases the dynamic range of the repressors, whereas the presence of repressors reduces the dynamic range of the activator. The reason for this is that bound activator assists in forming repressing O1O3 DNA loops. Fig 11A shows the gene expression normalized to the gene expression for the case of *R* = 0 (fold-change_R_)in the absence and presence of *A* = 0, *A* = 1000 activators respectively. The blue curve shows that in the absence of activator, repressors cause a decrease in the transcription-rate of approximately three orders of magnitude. However, the presence of activator may cause up to an additional single order of magnitude of decrease in the fold-change. While the net gene expression due to the presence of the activators remains higher, the presence of the activator causes a greater difference between the unrepressed and the repressed system. Fig 11B shows the gene expression normalized to the gene expression for the case of *A* = 0 (fold-change_A_) in the absence and presence of *R* = 0, *R* = 100 repressors respectively, illustrating that in the absence of repressor, the activators may cause up to a 200-fold-change in transcription rate, which drops down to ∼80-fold in the presence of a larger number of repressors.

This effect was experimentally observed by Kuhlman *et al.* [10], who measured the gene activity of the *lac* operon in *Escherichia coli* constructs that are unable to synthesize cAMP. Since CRP needs cAMP to activate the *lac* operon, the activating response to the cAMP-CRP complex could be induced externally. In the presence of cAMP in the growth medium, induction of the bacteria by IPTG (inactivating *lac* repressor) caused a >1600-fold-change in transcription levels. In the absence of cAMP, this fold-change dropped to only <250. Saiz and Vilar [61] also address this cooperative effect, which they term ‘robust expression with sensitive induction’.

Usually, a single copy of the *lac* operon exists in *E. coli* cells per chromosome and at slow growth rates the copy number of the *lac* operon is expected to be one or two. However, fast growing cells have multiple replication forks of the chromosome which can result in a higher copy numbers of the *lac* operon. Using this theory, we can calculate the effect of the existence of multiple gene copies without significant additional effort. Fig 10 (translucent blue surface) shows the fold-change of a *lac* operon regulated gene with a copy number of 10 in a single cell, as a function of activator and repressor numbers. At higher repressor concentrations there is no qualitative difference between this case and the single copy number case. At lower repressor concentrations, however, we find first a plateau in the fold-change, followed by a steep drop of over three orders of magnitude upon addition of one or two additional repressor molecules. The presence of multiple copies of the gene causes a titration effect in which the gene copies have to compete for the presence of LacI. The model shows clearly that in a competitive environment the interacting gene model presented here predicts a significantly different transcription rate than the isolated gene models.

**Fig 12:**
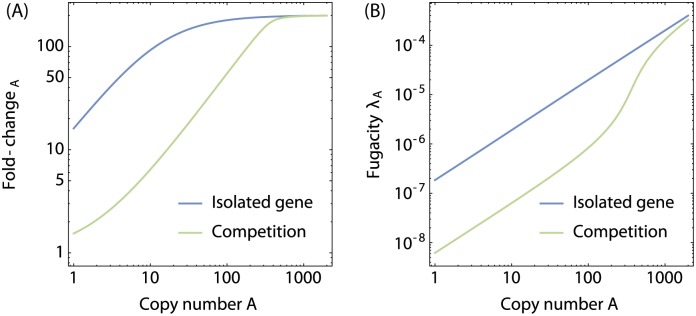
Effect of the competitive environment on activation. (**A**) Gene expression normalized to the gene expression at *A* = 0 (fold-change_A_) and (**B**) fugacity of activators, as a function of the number of activators in the isolated gene case (blue curves), and in the case where the activators are competed for by 350 additional competitor sites in the cell (green curves). In the interacting gene model the effective concentration of CRP is lower due to binding to competitor sites. Consequently, the transcription rate is significantly lower.

To illustrate this, we show in Fig 12 the fold-change_A_ of the *lac* operon as a function of the number of CRP in the cell in the case where the gene is isolated and when CRP is competed for by 350 competitor sites. CRP is strongly competed for in *E. coli* and consequently, the availability of CRP to bind to the *lac* operon is significantly lower than in the case where CRP has no other specific binding sites. The effect of competition on the transcription rate may exceed an order of magnitude.

## Conclusions and outlook

The rate of transcription initiation of a gene is strongly influenced by the competitive effects of the rest of the genome. The availability of transcription factors is dictated by the number and binding strength of competing binding sites, as well as the size of the non-specific reservoir. As was shown in Figs 10 and 12, competing binding sites can cause orders of magnitude changes in the transcription initiation rate. In the community of computational biology, theories for transcription initiation have been traditionally derived in the isolated gene limit [4, 7, 8], i.e. the transcription factors are not shared by multiple gene copies or competing binding sites in the cell. While these theories are successfully applied in that limit, competition for the molecules involved in transcription regulation is the rule, rather than the exception.

Attempts to include competition in the canonical ensemble have led to the use of combinatorics to keep track of the possible arrangements of transcription factors, as for example was successfully demonstrated by Burger *et al.* [32, 33], and Rydenfelt *et al.* [34]. The resulting expressions, however, do not lend themselves to intuitive interpretation.

The application of the grand canonical ensemble to the process of transcription initiation allows the native inclusion of competing binding sites. The reservoirs of binding sites are decoupled, so that one does not need to keep track of the individual arrangements of transcription factors in all the reservoirs simultaneously. Instead, the effect of multiple gene copies and other competing binding sites are embedded in the fugacity of the transcription factor.

The expressions we derived for the fold-change in transcription activity found in the grand canonical ensemble have the same intuitive form as in the case of an isolated gene. For each of the cases shown in Fig 6 the solution for fold-change calculated in the grand canonical ensemble can also be obtained by substituting *R*/*N*_ns_ by *λ*_R_, *A*/*N*_ns_ by *λ*_A_ and $R\left(R-1\right)/N_{\text{ns}}^{2}$ by $\lambda_{\text{R}}^{2}$ in the canonical solution. In each of the substitutions the transcription factor concentration is replaced by the appropriate fugacity *λ*_*i*_ (with *i* the kind of transcription factor), which can be interpreted as the effective concentration of the transcription factor in the presence of competing binding sites for that transcription factor. This correspondence suggests that the approach could be generalised with the help of automated computer scripts, such as has been done for their canonical counterparts (see e.g. Vilar [47]).

Competition in cells also manifests itself in the activation or inactivation of transcription factors by inducer molecules. In principle the effects of inducers can also be calculated using this theoretical framework by taking into account the different association states of the transcription factor-inducer equilibrium [64]. In a similar way, the formalism can be extended to include oligomerisation equilibria for transcription factors whose function depends on those details, a common scheme for global regulators in higher Eukaryotes [65]. The formalism developed here also holds promise in being able to compute protein-DNA binding in the context of high-throughput experiments such as Chip-Seq which explicitly examine the competition of different parts of the genome for the same proteins [66, 67].

## Supporting information

S1 Text RNAP fugacity. (PDF)

S2 Text Interactions in the non-specific reservoir. (PDF)

S3 Text Activator and repressor fugacity in the *lac* operon. (PDF)

S4 Text Weak promoter limit. (PDF)

S5 Text Relation between grand-canonical and canonical expressions. (PDF)
