# Supplementary material for: Self-consistent theory of transcriptional control in complex regulatory architectures
Source: PLoS One. 2017 Jul 7;12(7):e0179235. doi: 10.1371/journal.pone.0179235 (PMC5501422; doi:10.1371/journal.pone.0179235)
Supplement: S1 Text — (PDF) [file pone.0179235.s001.pdf]

S1 Text. RNAP fugacity

When calculating the fold-change we have thus far implicitly assumed that the RNAP fugacity does not change upon addition of transcription factors. This is not necessarily the case. To illustrate this, we will explicitly calculate the fugacity of RNAP in the presence (λ_P) and absence (λ_P^0) of transcription factors. We write

$$P = N\theta_\mathrm{P}(\lambda_\mathrm{P}, \lambda_\mathrm{R}) + N_\mathrm{ns}\theta_\mathrm{P}^\mathrm{ns}(\lambda_\mathrm{P}, \lambda_\mathrm{R}), \tag{S.1}$$

In the case of simple repression, the average occupation numbers can be found as

$$\left.\begin{aligned} \theta_\mathrm{P}(\lambda_\mathrm{P}, \lambda_\mathrm{R}) &= \frac{\lambda_\mathrm{P} x_\mathrm{P}}{1 + \lambda_\mathrm{P} x_\mathrm{P} + \lambda_\mathrm{R} x_\mathrm{R}} \simeq \frac{\lambda_\mathrm{P} x_\mathrm{P}}{1 + \lambda_\mathrm{R} x_\mathrm{R}}, \\ \theta_\mathrm{P}^\mathrm{ns}(\lambda_\mathrm{P}, \lambda_\mathrm{R}) &= \frac{\lambda_\mathrm{P}}{1 + \lambda_\mathrm{P}} \simeq \lambda_\mathrm{P}. \end{aligned}\right\} \tag{S.2}$$

Isolating λ_P from eq. (S.1), we obtain

$$\left.\begin{aligned} \lambda_\mathrm{P} &= \frac{P}{\frac{N}{1+\lambda_\mathrm{R} x_\mathrm{R}} x_\mathrm{P} + N_\mathrm{ns}}, \qquad \left(\simeq \frac{P}{N_\mathrm{ns}}\right) \\ \lambda_\mathrm{P}^0 &= \frac{P}{N x_\mathrm{P} + N_\mathrm{ns}}. \qquad \left(\simeq \frac{P}{N_\mathrm{ns}}\right) \end{aligned}\right\} \tag{S.3}$$

We write down the fraction $\lambda_\mathrm{P}/\lambda_\mathrm{P}^0$ as a series expansion.

$$\frac{\lambda_\mathrm{P}}{\lambda_\mathrm{P}^0} \simeq 1 + \frac{N x_\mathrm{P}}{N_\mathrm{ns}}\theta_\mathrm{R} - \left(\frac{N x_\mathrm{P}}{N_\mathrm{ns}}\right)^2 \frac{\theta_\mathrm{R}^2}{\lambda_\mathrm{R} x_\mathrm{R}} + \cdots \tag{S.4}$$

Since $\theta_\mathrm{R} \leq 1$, we see that $\lambda_\mathrm{P}/\lambda_\mathrm{P}^0$ becomes unity as long as $N x_\mathrm{P}/N_\mathrm{ns} \ll 1$. Typically, the number of non-specific sites is overwhelmingly large. In *E. coli*, N_ns is of the order 5×10^6 and $\epsilon_\mathrm{P} \sim -2.9 k_\mathrm{B}T$. This means that decoupling is justified for even large gene copy numbers, provided that $N \ll 3 \times 10^5$. Similarly, in activation architectures, we can write down a similar argument to show that the decoupling remains valid there. The RNAP fugacity in the case of simple activation becomes

$$\lambda_\mathrm{P} = \frac{P}{N\frac{(1+\lambda_\mathrm{A} x_\mathrm{A} x_\mathrm{AP})}{1+\lambda_\mathrm{A} x_\mathrm{A}} x_\mathrm{P} + N_\mathrm{ns}}, \qquad \text{(activation)} \tag{S.5}$$

which leads to the following series expansion

$$\frac{\lambda_\mathrm{P}}{\lambda_\mathrm{P}^0} = \begin{cases} 1 + \frac{N x_\mathrm{P}}{N_\mathrm{ns}}\theta_\mathrm{A}(1 - x_\mathrm{AP}) \\ -\left(\frac{N x_\mathrm{P}}{N_\mathrm{ns}}\right)^2 \theta_\mathrm{A}(1 - x_\mathrm{AP})\frac{1 + \lambda_\mathrm{A} x_\mathrm{A} x_\mathrm{AP}}{1 + \lambda_\mathrm{A} x_\mathrm{A}} + \cdots \end{cases} \tag{S.6}$$

This means that decoupling the RNAP fugacity is justified when $N x_\mathrm{P} \ll N_\mathrm{ns}$ and $N x_\mathrm{P}\theta_\mathrm{A}(1 - x_\mathrm{AP}) \ll N_\mathrm{ns}$. Since usually the number of non-specific sites in the genome of a cell is overwhelmingly large, the approximation is nearly always justified. In *E. coli*, this is the case when $N \ll 2 \times 10^3$.
