# Supplementary material for: Self-consistent theory of transcriptional control in complex regulatory architectures
Source: PLoS One. 2017 Jul 7;12(7):e0179235. doi: 10.1371/journal.pone.0179235 (PMC5501422; doi:10.1371/journal.pone.0179235)
Supplement: S2 Text — (PDF) [file pone.0179235.s002.pdf]

S2 Text. Interactions in the non-specific reservoir

The possibility remains that transcription factors and RNAP can interact with each other when both are bound to non-specific sites on the DNA. These interactions have thus far not been taken into account, since the local concentration of RNAP and transcription factor is low, due to the large number of non-specific sites. Here we will show how to include these interactions explicitly, and in which circumstances it is justified to neglect them.

We consider a single isolated non-specific site in a grand-canonical ensemble. The binding energies of RNAP and transcription factor are set to 0 as before. The grand-canonical partition function is then given by

$$\Xi_{\mathrm{ns}} = 1 + \lambda_{\mathrm{P}} + \lambda_{\mathrm{A}} + \lambda_{\mathrm{P}}\lambda_{\mathrm{A}}x_{\mathrm{AP}}, \tag{S.7}$$

where we have $\lambda_{\mathrm{P}}, \lambda_{\mathrm{A}}$ the fugacities of RNAP and activator respectively, and $x_{\mathrm{AP}} = \exp(-\beta\epsilon_{\mathrm{AP}})$ the gluelike interaction between RNAP and activator when both bound adjacent to each other. Note that, since the binding mode of transcription factors to non-specific DNA may be different to the binding mode to specific sites, conformational changes in the protein may also cause x_{AP} to be different from the activator-RNAP interaction on specific sites.

We calculate the occupation number of RNAP and activator on non-specific sites. For activators, this becomes

$$\theta_{\mathrm{A}}^{\mathrm{ns}} = \frac{\lambda_{\mathrm{A}}(1+\lambda_{\mathrm{P}}x_{\mathrm{AP}})}{1+\lambda_{\mathrm{P}}+\lambda_{\mathrm{A}}+\lambda_{\mathrm{P}}\lambda_{\mathrm{A}}x_{\mathrm{AP}}} \simeq \lambda_{\mathrm{A}}(1+\lambda_{\mathrm{P}}x_{\mathrm{AP}}), \qquad (\lambda_{\mathrm{P}}, \lambda_{\mathrm{A}} \ll 1) \tag{S.8}$$

When deriving eq. (29), we assumed that $\lambda_{\mathrm{P}}x_{\mathrm{P}}x_{\mathrm{AP}} \ll 1$. Since the binding energy of RNAP to specific sites is more favourable than to non-specific sites, $x_{\mathrm{P}} > 1$, the assumption $\lambda_{\mathrm{P}}x_{\mathrm{AP}} \ll 1$ is already taken care of (provided x_{AP} is not significantly different on non-specific sites than on specific sites). In that case, we have $\theta_{\mathrm{A}}^{\mathrm{ns}} = \lambda_{\mathrm{A}}$ as before.

For RNAP, the occupation number becomes

$$\theta_{\mathrm{P}}^{\mathrm{ns}} = \frac{\lambda_{\mathrm{P}}(1+\lambda_{\mathrm{A}}x_{\mathrm{AP}})}{1+\lambda_{\mathrm{A}}+\lambda_{\mathrm{P}}+\lambda_{\mathrm{P}}\lambda_{\mathrm{A}}x_{\mathrm{AP}}} \simeq \lambda_{\mathrm{P}}(1+\lambda_{\mathrm{A}}x_{\mathrm{AP}}), \qquad (\lambda_{\mathrm{P}}, \lambda_{\mathrm{A}} \ll 1) \tag{S.9}$$

In this situation, eq. (S.5) becomes

$$\lambda_{\mathrm{P}} = \frac{P}{N\frac{(1+\lambda_{\mathrm{A}}x_{\mathrm{A}}x_{\mathrm{AP}})}{1+\lambda_{\mathrm{A}}x_{\mathrm{A}}}x_{\mathrm{P}} + N_{\mathrm{ns}}(1+\lambda_{\mathrm{A}}x_{\mathrm{AP}})}, \qquad \text{(activation)} \tag{S.10}$$

The zeroth order term in the series expansion of $\lambda_{\mathrm{P}}/\lambda_{\mathrm{P}}^{0}$, eq. (S.6), now does not become unity, rather, it becomes $(1+\lambda_{\mathrm{A}}x_{\mathrm{AP}})^{-1}$. Usually, $\lambda_{\mathrm{A}} \ll 1$, but on specific sites, ϵ_{AP} can be as high as $-5k_{\mathrm{B}}T$, leading to $x_{\mathrm{AP}} \sim 200$. In the situation that λ_{A} is comparatively high on the order of $\sim 10^{-3}$, which is the case when thousands of activators are present in the cell, we can not make the assumption $\lambda_{\mathrm{A}}x_{\mathrm{AP}} \ll 1$ anymore and we have to explicitly take into account that $\lambda_{\mathrm{P}}/\lambda_{\mathrm{P}}^{0} \simeq (1+\lambda_{\mathrm{A}}x_{\mathrm{AP}})^{-1}$.
