# Supplementary material for: Self-consistent theory of transcriptional control in complex regulatory architectures
Source: PLoS One. 2017 Jul 7;12(7):e0179235. doi: 10.1371/journal.pone.0179235 (PMC5501422; doi:10.1371/journal.pone.0179235)
Supplement: S3 Text — (PDF) [file pone.0179235.s003.pdf]

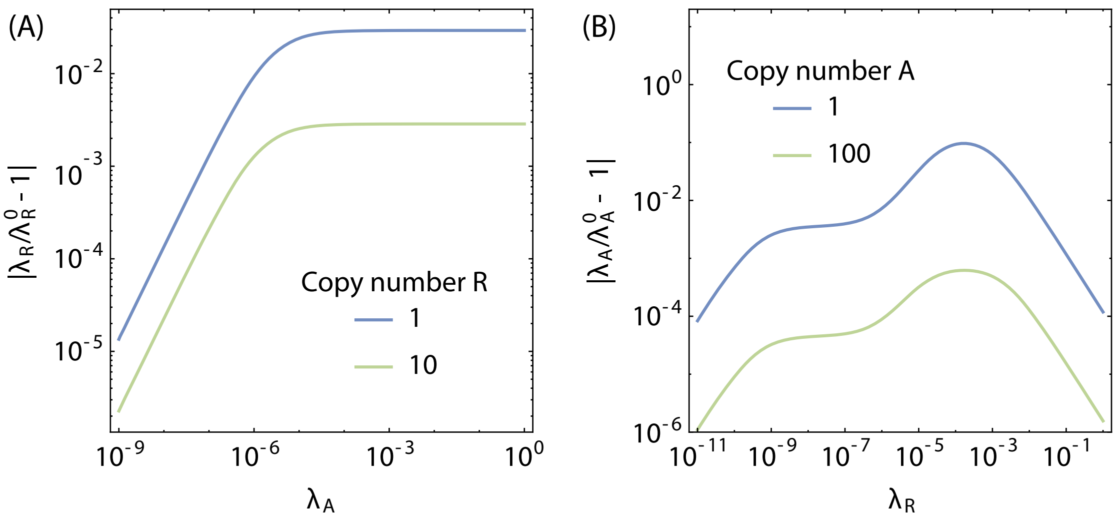

Figure S1. Deviation of the unperturbed transcription factor fugacity from the real transcription factor fugacity in the *lac* operon.
(**A**) Relative deviation of the unperturbed activator fugacity λ_A^0 from the real activator fugacity λ_A as a function of repressor fugacity. (**B**) Relative deviation of the unperturbed repressor fugacity λ_R^0 from the real repressor fugacity λ_R as a function of activator fugacity. Especially the unperturbed activator fugacity may deviate significantly from the real activator fugacity when the total number of activators is low.

S3 Text. Activator and repressor fugacity in the *lac* operon

Fig. 8 shows that the fugacities of repressor and activator do not noticably change when the other transcription factor is present. However, there is a small effect, the magnitude of which depends on the number of transcription factors and competing binding sites. Here we explicitly plot $|\lambda_A/\lambda_A^0 - 1|$ for activators in the presence of a given number of repressors (Fig. S1A). For repressors, we plot $|\lambda_R/\lambda_R^0 - 1|$ in the presence of a given number of activators (Fig. S1B). When the quantity $|\lambda/\lambda^0 - 1|$ drops, the unperturbed transcription factor fugacity becomes asymptotically equal to the real fugacity of the transcription factor in the presence of the other transcription factor. As can be seen from Fig. S1, for low copy number of transcription factor, there are certain regimes where the unperturbed fugacity deviates from the real fugacity. For activators, this effect is stronger, especially when there are no competing CRP binding sites available. This suggests that the easiest way to decouple the activator and repressor fugacity is by making the assumption that $\lambda_P \approx \lambda_P^0$, where λ_P^0 is the fugacity of repressor in the absence of any activators (blue curve in Fig. 8B), and calculating λ_A as a function of λ_R^0. This is the method we have adopted in this work.

Alternatively, since the change in fugacity of either kind of transcription factor varies only very weakly with the fugacity of the other transcription factor, one could calculate λ_P^0 and λ_A^0 as an initial guess, with which the other transcription factor fugacity could be calculated. An extension of this would be to set up an iterative, self-consistent approach where calculation of activator fugacity could be used to refine the calculation of the repressor fugacity, which in turn could be used for further refinement until self-consistent values for λ_A and λ_R were found.
