# Supplementary material for: Self-consistent theory of transcriptional control in complex regulatory architectures
Source: PLoS One. 2017 Jul 7;12(7):e0179235. doi: 10.1371/journal.pone.0179235 (PMC5501422; doi:10.1371/journal.pone.0179235)
Supplement: S4 Text — (PDF) [file pone.0179235.s004.pdf]

S4 Text. Weak promoter limit

We have so far usually worked in the weak promoter limit, i.e. $\lambda_{\mathrm{P}} x_{\mathrm{P}} \ll 1$. Where the regulatory architecture leads to activation, this assumption on its own is not enough to make the fold-change independent of RNAP fugacity. For that reason, we have invoked the assumption that $\lambda_{\mathrm{P}} x_{\mathrm{P}} \ll \Sigma_0/\Sigma_{\mathrm{P}}$, with $\Sigma_0, \Sigma_{\mathrm{P}}$ defined above, where, conveniently enough, it proves to be the case that this fraction is equal to 1/fold-change, provided that we may actually make this assumption. The fold-change calculated thus provides us with a convenient tool to check a posteriori whether this assumption is justified.

A typical binding energy of RNAP to a promoter is $\epsilon_{\mathrm{P}} \sim -2.9 k_{\mathrm{B}} T$ (*E. coli* RNAP to *lac* promoter). In *E. coli*, there are typically ~ 1000 RNAP molecules in a single cell, leading to an RNAP fugacity of $\lambda_{\mathrm{P}} \approx P/N_{\mathrm{ns}} \sim 10^{-4}$. This means that typically, $\lambda_{\mathrm{P}} x_{\mathrm{P}} \sim 2 \times 10^{-3}$. In order for the assumption to hold, we need to have $\Sigma_{\mathrm{P}}/\Sigma_0 \ll 5 \times 10^2$. If not, then the assumption breaks down and the RNAP fugacity needs to be calculated explicitly in order to calculate an accurate fold-change.

In Fig. 10 is plotted the fold-change of the *E. coli lac* operon as a function of the total number of CRP (activators) and *lac* repressors. We see that the fold-change never exceeds 10^2, even for very high number of activators. The activator binding sites are essentially saturated with activators. In this regime, $\Sigma_{\mathrm{P}}/\Sigma_0$ remains lower than 5×10^2, although it does come close. This situation, however, only occurs when close to no repressors are present in the cell. When just over a single repressor is present, the fold-change drops dramatically to well below 1. In those circumstances, the assumption that $\lambda_{\mathrm{P}} x_{\mathrm{P}} \ll \Sigma_0/\Sigma_{\mathrm{P}}$ is already taken care of by the weak promoter limit.
