# Supplementary material for: Self-consistent theory of transcriptional control in complex regulatory architectures
Source: PLoS One. 2017 Jul 7;12(7):e0179235. doi: 10.1371/journal.pone.0179235 (PMC5501422; doi:10.1371/journal.pone.0179235)
Supplement: S5 Text — (PDF) [file pone.0179235.s005.pdf]

S5 Text. Relation between grand-canonical and canonical expressions

This section is to show the relation between the canonical and grand-canonical probability of finding RNAP bound to the promoter, and to show in which conditions the two are equivalent. We will derive an expression for the occupation number of RNAP bound to the promoter site of a gene in the two ensembles and show that the two expressions have the same form. The fugacity will play the role of an effective available concentration in the grand-canonical ensemble, while the same role in the canonical ensemble will be played by a factor derived from combinatorial arguments, yet still behaving as an effective available concentration. Moreover, we will show that the two are asymptotically equal to each other in the thermodynamic limit, showing that in those circumstances the two ensembles become equivalent.

Canonical ensemble We start in the canonical ensemble, where we consider a single gene that can bind RNAP (of which there are P molecules in the cell), and a number of transcription factors $\mathrm{A}, \mathrm{B}, \dots$ of copy number $A, B, \dots$ respectively. We set the effective energy of non-specific sites to 0 and consider only the binding energies of RNAP and transcription factors to their specific sites on the DNA, and interaction energies between specifically bound RNAP and transcription factors.

We do not explicitly specify the number of operator sites a transcription factor has on a specific gene, it can be 0, 1 or more. If a gene has more than one site for a single transcription factor, then of course there are multiple possibilities of binding the transcription factors to these sites with the same occupation numbers. We therefore define $Z(p, a, b, \dots)$ as the sum of the Boltzmann-factors $\exp(-\beta\epsilon_i(p, a, b, \dots))$ for each adsorption state i that has $p, a, b, \dots$ number of molecules of $\mathrm{RNAP}, \mathrm{A}, \mathrm{B}, \dots$ bound specifically to the gene, respectively.

$$Z(p, a, b, \dots) \equiv \sum_i \exp(-\beta\epsilon_i(p, a, b, \dots)), \tag{S.11}$$

The RNAP molecules and transcription factors that are not bound to a specific site are distributed over the non-specific sites of the DNA. The number of ways to distribute these molecules over the non-specific DNA sites is given by the multinomial coefficient

$$\binom{N_{\mathrm{ns}}}{P, A, B, \dots} = \frac{N_{\mathrm{ns}}!}{P!A!B!\cdots(N_{\mathrm{ns}} - P - A - B - \cdots)!}. \tag{S.12}$$

When a transcription factor binds to a specific site in the gene, it is removed from the non-specific sites, so we remove it from the multinomial factor. For example, if a single molecule of A binds to its specific site, the number of ways the remainder of RNAP and transcription factor molecules can be distributed over the non-specific DNA is given by

$$\binom{N_{\mathrm{ns}}}{P, A-1, B, \dots}. \tag{S.13}$$

The total weight of the configuration state that has a single molecule of A bound to the gene is then given by the product of the multinomial factor and Z. Thus,

$$Z_{\mathrm{state}}(0, 1, 0, \dots) = \binom{N_{\mathrm{ns}}}{P, A-1, B, \dots} Z(0, 1, 0, \dots). \tag{S.14}$$

We are not interested in the internal degrees of freedom the different species in the system have — these do not change upon specific binding of RNAP or transcription factors to the gene, and therefore only attribute a constant factor in the partition

function of the system. The only configurational states that we are interested in, are those states that differ in the number of RNAP, A, B, ... bound specifically to the gene. We find the total effective partition function of the genome by summing Z_{state} over all these states consistent with $p, a, b, \ldots$ number of molecules of RNAP, A, B, ... bound specifically.

$$
\begin{aligned}
Z_{\text{tot}} &= \sum_{p=0}^{1} \sum_{a} \sum_{b} \cdots Z_{\text{state}}(p, a, b, \ldots) \\
&= \sum_{p=0}^{1} \sum_{a} \sum_{b} \cdots \binom{N_{\text{ns}}}{P-p, A-a, B-b, \ldots} Z(p, a, b, \ldots). \qquad \text{(S.15)}
\end{aligned}
$$

It will turn out to be useful to isolate the occupation numbers $p, a, b, \ldots$ from the multinomial factor, so that it becomes a constant that depends only on the total number of molecules. We do this by considering the definition of the multinomial coefficient eq. (S.12)

$$
\begin{aligned}
\binom{N_{\text{ns}}}{P-p, A-a, B-b, \ldots} = \binom{N_{\text{ns}}}{P, A, B, \ldots} & \frac{(N_{\text{ns}} - P - A - B - \cdots)!}{(N_{\text{ns}} - P + p - A + a - B + b - \cdots)!} \\
& \times \frac{P!}{(P-p)!} \frac{A!}{(A-a)!} \frac{B!}{(B-b)!} \cdots \qquad \text{(S.16)}
\end{aligned}
$$

The first factor is now a multinomial coefficient that is constant and depends only on the total number of RNAP and transcription factors. The second factor still depends on the occupation numbers $p, a, b, \ldots$, but when N_{ns} is sufficiently large, that is, when $N_{\text{ns}} \gg P - p + A - a + B - b + \cdots$, we can apply the following approximation.

$$
\frac{(N_{\text{ns}} - P - A - B - \cdots)!}{(N_{\text{ns}} - P + p - A + a - B + b - \cdots)!} \simeq N_{\text{ns}}^{-(p+a+b+\cdots)}. \qquad \text{(S.17)}
$$

Substituting eqs. (S.16) and (S.17) into eq. (S.15), we obtain

$$
Z_{\text{tot}} = \binom{N_{\text{ns}}}{P, A, B, \ldots} \sum_{p=0}^{1} \sum_{a} \sum_{b} \cdots \frac{P!}{(P-p)! N_{\text{ns}}^{p}} \frac{A!}{(A-a)! N_{\text{ns}}^{a}} \frac{B!}{(B-b)! N_{\text{ns}}^{b}} \cdots Z(p, a, b, \ldots). \qquad \text{(S.18)}
$$

We have now removed the multinomial coefficient from the sum and grouped all factors that are related to RNAP, A, B, ... This expression for the canonical partition function shows us that the weight of a specific configurational state is given by the Boltzmann weight of the energy of the state, multiplied by a corrective factor that takes into account the redistribution of the remaining molecules on the DNA, and that this corrective factor behaves as an effective available concentration per specifically adsorbed molecule. We will discuss this role later on.

To find the occupation number of RNAP bound to the promoter site, we will explicitly write out the first sum as

$$
\begin{aligned}
Z_{\text{tot}} = \binom{N_{\text{ns}}}{P, A, B, \ldots} \Bigg[& \sum_{a} \sum_{b} \cdots \frac{A!}{(A-a)! N_{\text{ns}}^{a}} \frac{B!}{(B-b)! N_{\text{ns}}^{b}} \cdots Z(0, a, b, \ldots) \\
& + \frac{P}{N_{\text{ns}}} \sum_{a} \sum_{b} \cdots \frac{A!}{(A-a)! N_{\text{ns}}^{a}} \frac{B!}{(B-b)! N_{\text{ns}}^{b}} \cdots Z(1, a, b, \ldots) \Bigg]. \qquad \text{(S.19)}
\end{aligned}
$$

The occupation number of RNAP being bound to the promoter can then be written as

$$\theta_{\mathrm{P}}(P, A, B, \dots) = \frac{\dfrac{P}{N_{\mathrm{ns}}} \sum_a \sum_b \cdots \dfrac{A!}{(A-a)! N_{\mathrm{ns}}^a} \dfrac{B!}{(B-b)! N_{\mathrm{ns}}^b} \cdots Z(1, a, b, \dots)}{\sum_{p=0}^{1} \sum_a \sum_b \cdots \dfrac{P!}{(P-p)! N_{\mathrm{ns}}^p} \dfrac{A!}{(A-a)! N_{\mathrm{ns}}^a} \dfrac{B!}{(B-b)! N_{\mathrm{ns}}^b} \cdots Z(p, a, b, \dots)}, \tag{S.20}$$

where the multinomial factor was a common factor in both the denominator and enumerator, and cancels out. This expression is the main canonical result, and we will see that the equivalent equation in the grand-canonical ensemble has an identical form. For now, we can continue along this path and derive a general expression for the canonical fold-change. For this, it is easiest to introduce a shorthand

$$\begin{aligned} \Sigma_{\mathrm{P}}^{\mathrm{c}} &\equiv \sum_a \sum_b \cdots \frac{A!}{(A-a)! N_{\mathrm{ns}}^a} \frac{B!}{(B-b)! N_{\mathrm{ns}}^b} \cdots Z(1, a, b, \dots)/x_{\mathrm{P}} \\ \Sigma_0^{\mathrm{c}} &\equiv \sum_a \sum_b \cdots \frac{A!}{(A-a)! N_{\mathrm{ns}}^a} \frac{B!}{(B-b)! N_{\mathrm{ns}}^b} \cdots Z(0, a, b, \dots), \end{aligned} \tag{S.21}$$

where $x_{\mathrm{P}} = \exp -\beta\epsilon_{\mathrm{P}}$, and the superscript c denotes that this is the canonical result. Using the shorthand, we can write

$$\theta_{\mathrm{P}}(P, A, B, \dots) = \frac{\dfrac{P}{N_{\mathrm{ns}}} x_{\mathrm{P}} \Sigma_{\mathrm{P}}^{\mathrm{c}}}{\dfrac{P}{N_{\mathrm{ns}}} x_{\mathrm{P}} \Sigma_{\mathrm{P}}^{\mathrm{c}} + \Sigma_0^{\mathrm{c}}}. \tag{S.22}$$

The fold-change is equal to $\theta_{\mathrm{P}}(P, A, B, \dots)/\theta_{\mathrm{P}}(P, 0, 0, \dots)$. When $A = B = \cdots = 0$, we can see from eq. (S.21) that $\Sigma_{\mathrm{P}}^{\mathrm{c}} = Z(1, 0, 0, \dots)/x_{\mathrm{P}} = 1$ and $\Sigma_0^{\mathrm{c}} = Z(0, 0, 0, \dots) = 1$. Consequently,

$$\theta_{\mathrm{P}}(P, 0, 0, \dots) = \frac{\dfrac{P}{N_{\mathrm{ns}}} x_{\mathrm{P}}}{1 + \dfrac{P}{N_{\mathrm{ns}}} x_{\mathrm{P}}} \approx \frac{P}{N_{\mathrm{ns}}} x_{\mathrm{P}}, \qquad \left(\frac{P}{N_{\mathrm{ns}}} x_{\mathrm{P}} \ll 1\right) \tag{S.23}$$

The approximated expression is valid in the weak promoter limit. The fold-change is then found by dividing eq. (S.22) by eq. (S.23).

$$\text{Fold-change} = \frac{\Sigma_{\mathrm{P}}^{\mathrm{c}}}{\dfrac{P}{N_{\mathrm{ns}}} x_{\mathrm{P}} \Sigma_{\mathrm{P}}^{\mathrm{c}} + \Sigma_0^{\mathrm{c}}}. \tag{S.24}$$

We make one further approximation, namely

$$\text{Fold-change} \simeq \frac{\Sigma_{\mathrm{P}}^{\mathrm{c}}}{\Sigma_0^{\mathrm{c}}}, \qquad \left(\frac{P}{N_{\mathrm{ns}}} x_{\mathrm{P}} \ll \frac{\Sigma_0^{\mathrm{c}}}{\Sigma_{\mathrm{P}}^{\mathrm{c}}}\right) \tag{S.25}$$

In the case of repressive regulatory scenarios, the fraction $\Sigma_0^{\mathrm{c}}/\Sigma_{\mathrm{P}}^{\mathrm{c}} > 1$, which means that this condition is already taken care of by the weak promoter limit that we imposed in eq. (S.23). For all activating scenarios, eq. (S.25) will still work, provided we can assume that $P x_{\mathrm{P}}/N_{\mathrm{ns}} \ll \Sigma_0^{\mathrm{c}}/\Sigma_{\mathrm{P}}^{\mathrm{c}}$, which is in those cases not automatically taken care of by the weak promoter limit. As discussed above, the fact that $\Sigma_0^{\mathrm{c}}/\Sigma_{\mathrm{P}}^{\mathrm{c}} \simeq 1/\text{fold-change}$, we can use the fold-change as a convenient tool to verify this assumption *a posteriori*.

Grand-canonical ensemble We now turn to the grand-canonical ensemble. We now consider the situation in one of the N gene copies, and the non-specific sites and competing sites are included as additional reservoirs with which our system is in contact. If the gene is present at higher copy number, then there are simply multiple independent copies of this system. The gene copies are decoupled from each other and the rest of the genome, thus effectively eliminating the constraint on the total number of $\mathrm{RNAP}, \mathrm{A}, \mathrm{B}, \dots$. Consequently, the weight of each state isn't dependent on the combinatorial problem of how to distribute the remaining transcription factors over the non-specific DNA, but on $\lambda = \exp(\beta\mu)$ of each species. The factor λ will mathematically act as a Lagrange multiplier for the constraint on the total number of molecules, but also has the physical meaning of fugacity or activity, being an effective concentration.

The grand canonical partition function of a single gene is given by

$$\Xi = \sum_{p=0}^{1} \sum_{a} \sum_{b} \cdots \lambda_{\mathrm{P}}^{p} \lambda_{\mathrm{A}}^{a} \lambda_{\mathrm{B}}^{b} \cdots Z(p, a, b, \dots), \tag{S.26}$$

where we have $\lambda_{\mathrm{P}}, \lambda_{\mathrm{A}}, \lambda_{\mathrm{B}}, \dots$ the fugacities of RNAP and species $\mathrm{A}, \mathrm{B}, \dots$ respectively. The factor $Z(p, a, b, \dots)$ is the same as above. Comparing eqs. (S.18) and (S.26), we can immediately see the similarities between the two expressions. In both ensembles, we sum over the different occupation numbers of RNAP and transcription factors bound specifically to the gene, and in both cases the weight of each state is given by the product of the Boltzmann factors and an expression that acts as an effective available concentration per specifically adsorbed molecule. Of course, the canonical expression also has a multinomial coefficient that is absent from the grand-canonical expression, since the grand-canonical system is decoupled from the rest of the genome. To find the grand-canonical occupation number of RNAP bound to the promoter, we will also write out the first sum explicitly.

$$\begin{aligned}\Xi = &\sum_{a} \sum_{b} \cdots \lambda_{\mathrm{A}}^{a} \lambda_{\mathrm{B}}^{b} \cdots Z(0, a, b, \dots) \\ &+ \lambda_{\mathrm{P}} \sum_{a} \sum_{b} \cdots \lambda_{\mathrm{A}}^{a} \lambda_{\mathrm{B}}^{b} \cdots Z(1, a, b, \dots).\end{aligned} \tag{S.27}$$

We can write down the occupation number of RNAP bound to the promoter.

$$\theta_{\mathrm{P}}(\lambda_{\mathrm{P}}, \lambda_{\mathrm{A}}, \lambda_{\mathrm{B}}, \dots) = \frac{\lambda_{\mathrm{P}} \sum_{a} \sum_{b} \cdots \lambda_{\mathrm{A}}^{a} \lambda_{\mathrm{B}}^{b} \cdots Z(1, a, b, \dots)}{\sum_{p=0}^{1} \sum_{a} \sum_{b} \cdots \lambda_{\mathrm{P}}^{p} \lambda_{\mathrm{A}}^{a} \lambda_{\mathrm{B}}^{b} \cdots Z(p, a, b, \dots)}. \tag{S.28}$$

By comparing eqs. (S.20) and (S.28), we see that the expressions for θ_{P} in both ensembles are equimorphous when we make the substitutions

$$\lambda_{\mathrm{X}}^{x} \leftrightarrow \frac{X!}{(X - x)! N_{\mathrm{ns}}^{x}}, \tag{S.29}$$

for all involved species X where $\mathrm{X} = \mathrm{RNAP}, \mathrm{A}, \mathrm{B}, \dots$. Explicitly, this means that we can use the following substitutions for different powers of λ_{X}

$$\lambda_{\mathrm{X}}^{0} \leftrightarrow 1 \qquad \lambda_{\mathrm{X}}^{1} \leftrightarrow \frac{X}{N_{\mathrm{ns}}} \qquad \lambda_{\mathrm{X}}^{2} \leftrightarrow \frac{X}{N_{\mathrm{ns}}} \frac{X - 1}{N_{\mathrm{ns}}} \qquad \dots \tag{S.30}$$

From the equivalence of the expressions for θ_{P} in the canonical and grand-canonical ensemble, we see that we can go from the expression in one ensemble to the other ensemble using the substitutions in eq. (S.29). Otherwise, the expressions are completely identical, regardless of the regulatory architecture.

We continue to find a general expression for fold-change in the grand-canonical ensemble, that are comparable to their canonical analogs. We introduce the shorthands

$$\begin{aligned}\Sigma_{\mathrm{P}}^{\mathrm{gc}} &\equiv \sum_a \sum_b \cdots \lambda_{\mathrm{A}}^a \lambda_{\mathrm{B}}^b \cdots Z(1, a, b, \dots)/x_{\mathrm{P}} \\ \Sigma_0^{\mathrm{gc}} &\equiv \sum_a \sum_b \cdots \lambda_{\mathrm{A}}^a \lambda_{\mathrm{B}}^b \cdots Z(0, a, b, \dots)\end{aligned} \tag{S.31}$$

where $x_{\mathrm{P}} = \exp(-\beta\epsilon_{\mathrm{P}})$ and the superscript gc denotes that this is the grand-canonical result. These can be used to write eq. (S.28) as

$$\theta_{\mathrm{P}}(\lambda_{\mathrm{P}}, \lambda_{\mathrm{A}}, \lambda_{\mathrm{B}}, \dots) = \frac{\lambda_{\mathrm{P}} x_{\mathrm{P}} \Sigma_{\mathrm{P}}^{\mathrm{gc}}}{\Sigma_0^{\mathrm{gc}} + \lambda_{\mathrm{P}} x_{\mathrm{P}} \Sigma_{\mathrm{P}}^{\mathrm{gc}}} \tag{S.32}$$

In the absence of transcription factors, the expression for θ_{P} becomes really simple

$$\theta_{\mathrm{P}}(\lambda_{\mathrm{P}}, 0, 0, \dots) = \frac{\lambda_{\mathrm{P}} x_{\mathrm{P}}}{1 + \lambda_{\mathrm{P}} x_{\mathrm{P}}} \simeq \lambda_{\mathrm{P}} x_{\mathrm{P}}, \qquad (\lambda_{\mathrm{P}} x_{\mathrm{P}} \ll 1) \tag{S.33}$$

To calculate the fold-change, we divide the two and obtain

$$\text{Fold-change} = \frac{\Sigma_{\mathrm{P}}^{\mathrm{gc}}}{\Sigma_0^{\mathrm{gc}} + \lambda_{\mathrm{P}} x_{\mathrm{P}} \Sigma_{\mathrm{P}}^{\mathrm{gc}}} \simeq \frac{\Sigma_{\mathrm{P}}^{\mathrm{gc}}}{\Sigma_0^{\mathrm{gc}}}, \qquad \left(\lambda_{\mathrm{P}} x_{\mathrm{P}} \ll \frac{\Sigma_0^{\mathrm{gc}}}{\Sigma_{\mathrm{P}}^{\mathrm{gc}}}\right) \tag{S.34}$$

Here we have made essentially the same assumption as in eq. (S.25), valid for repressive scenarios in the weak promoter limit, while for activating scenarios it becomes the strictest assumption.

The canonical and grand canonical expressions for the fold-change, eqs. (S.25) and (S.34), both have the same dependence on $\Sigma_{\mathrm{P}}^{\mathrm{c,gc}}, \Sigma_0^{\mathrm{c,gc}}$, and we can see from their definitions, eqs. (S.21) and (S.31) that the only difference between the canonical and grand-canonical fold-change is the substitution of $X!/(X-x)!N_{\mathrm{ns}}^x$ in the canonical result for λ_{x} in the grand-canonical result, for all involved molecules $\mathrm{RNAP, A, B, \dots}$ that have a binding site on the gene.

As an example, for simple repression, the canonical $\Sigma_0^{\mathrm{c}}, \Sigma_{\mathrm{P}}^{\mathrm{c}}$ are given by

$$\begin{aligned}\Sigma_{\mathrm{P}}^{\mathrm{c}} &= \sum_{r=0}^{1} \frac{R!}{(R-r)!N_{\mathrm{ns}}^r} \frac{Z(1,r)}{x_{\mathrm{P}}} = \frac{x_{\mathrm{P}}}{x_{\mathrm{P}}} = 1, \\ \Sigma_0^{\mathrm{c}} &= \sum_{r=0}^{1} \frac{R!}{(R-r)!N_{\mathrm{ns}}^r} Z(0,r) = 1 + \frac{R}{N_{\mathrm{ns}}} x_{\mathrm{R}}.\end{aligned} \tag{S.35}$$

The canonical fold-change is then given by $\Sigma_{\mathrm{P}}^{\mathrm{c}}/\Sigma_0^{\mathrm{c}} = (1 + (R/N_{\mathrm{ns}})x_{\mathrm{R}})^{-1}$, as was determined earlier [1]. In the grand canonical ensemble, we have

$$\begin{aligned}\Sigma_{\mathrm{P}}^{\mathrm{gc}} &= \sum_{r=0}^{1} \lambda_{\mathrm{R}}^r \frac{Z(1,r)}{x_{\mathrm{P}}} = \frac{x_{\mathrm{P}}}{x_{\mathrm{P}}} = 1, \\ \Sigma_0^{\mathrm{gc}} &= \sum_{r=0}^{1} \lambda_{\mathrm{R}}^r Z(0,r) = 1 + \lambda_{\mathrm{R}} x_{\mathrm{R}},\end{aligned} \tag{S.36}$$

which leads to the fold-change, in the form derived earlier in this article, $\Sigma_{\mathrm{P}}^{\mathrm{gc}}/\Sigma_0^{\mathrm{gc}} = (1 + \lambda_{\mathrm{R}} x_{\mathrm{R}})^{-1}$.

Ensemble equivalence In actual cells the number of transcription factors can be as small as ten. With such small numbers, ensemble equivalence is an issue and we address it here. While the two ensembles are not identical, we see that the canonical and grand-canonical expressions for θ_{P}, as well as for the fold-change have essentially the same form, where eq. (S.29) identifies the substitutions that make the expressions equal.

In the thermodynamic limit, when $X \gg 1$ for a species $\mathrm{X} = \mathrm{RNAP}, \mathrm{A}, \mathrm{B}, \ldots$ (but $X \ll N_{\mathrm{ns}}$), we see that the canonical expression in eq. (S.29) simplifies to

$$\frac{X!}{(X-x)!N_{\mathrm{ns}}^x} \simeq \frac{X^x}{N_{\mathrm{ns}}^x}, \qquad (1 \ll X \ll N_{\mathrm{ns}}) \tag{S.37}$$

Here, X is the number of molecules of species $\mathrm{X} = \mathrm{RNAP}, \mathrm{A}, \mathrm{B}, \ldots$, with x the number of X adsorbed to the gene in the state we're interested in. For the grand-canonical ensemble we first consider the reservoir of non-specific sites. The expected number of molecules of X bound to a non-specific site $\langle X \rangle$ is given by

$$\langle X \rangle = N_{\mathrm{ns}} p_{\mathrm{bound,ns}} = N_{\mathrm{ns}} \frac{\lambda_{\mathrm{X}}}{1+\lambda_{\mathrm{X}}} \simeq N_{\mathrm{ns}} \lambda_{\mathrm{X}}, \qquad (\lambda_{\mathrm{X}} \ll 1) \tag{S.38}$$

as we set the binding energy of non-specific sites to 0. Rewriting

$$\lambda_{\mathrm{X}} = \frac{\langle X \rangle}{N_{\mathrm{ns}}} \tag{S.39}$$

When X is sufficiently large, the average number of X bound to non-specific sites becomes equal to the total number of X in the cell. In this limit, we can see that the substitution in eq. (S.29) becomes exact and that

$$\lambda_{\mathrm{X}}^x \simeq \frac{X!}{(X-x)!N_{\mathrm{ns}}^x} \simeq \frac{X^x}{N_{\mathrm{ns}}^x}, \qquad \text{(Thermodynamic limit)} \tag{S.40}$$

References

1. Phillips R, Kondev J, Theriot J, Garcia HG. Physical biology of the cell. 2nd ed. New York: Garland Science; 2012.
